# Ecotin, a microbial inhibitor of serine proteases, blocks multiple complement dependent and independent microbicidal activities of human serum

**DOI:** 10.1371/journal.ppat.1008232

**Published:** 2019-12-20

**Authors:** Zoltán Attila Nagy, Dávid Szakács, Eszter Boros, Dávid Héja, Eszter Vígh, Noémi Sándor, Mihály Józsi, Gábor Oroszlán, József Dobó, Péter Gál, Gábor Pál

**Affiliations:** 1 Department of Biochemistry, ELTE, Eötvös Loránd University, Budapest, Hungary; 2 Department of Nephrology, Icahn School of Medicine at Mount Sinai, New York, New York, United States of America; 3 Department of Immunology, ELTE, Eötvös Loránd University, Budapest, Hungary; 4 Institute of Enzymology, Research Centre for Natural Sciences, Hungarian Academy of Sciences, Budapest, Hungary; University of Birmingham, UNITED KINGDOM

## Abstract

Ecotin is a serine protease inhibitor produced by hundreds of microbial species, including pathogens. Here we show, that ecotin orthologs from *Escherichia coli*, *Yersinia pestis*, *Pseudomonas aeruginosa* and *Leishmania major* are potent inhibitors of MASP-1 and MASP-2, the two key activator proteases of the complement lectin pathway. Factor D is the key activator protease of another complement activation route, the alternative pathway. We show that ecotin inhibits MASP-3, which is the sole factor D activator in resting human blood. In pathway-specific ELISA tests, we found that all ecotin orthologs are potent lectin pathway inhibitors, and at high concentration, they block the alternative pathway as well. In flow cytometry experiments, we compared the extent of complement-mediated opsonization and lysis of wild-type and ecotin-knockout variants of two *E*. *coli* strains carrying different surface lipopolysaccharides. We show, that endogenous ecotin provides significant protections against these microbicidal activities for both bacteria. By using pathway specific complement inhibitors, we detected classical-, lectin- and alternative pathway-driven complement attack from normal serum, with the relative contributions of the activation routes depending on the lipopolysaccharide type. Moreover, in cell proliferation experiments we observed an additional, complement-unrelated antimicrobial activity exerted by heat-inactivated serum. While ecotin-knockout cells are highly vulnerable to these activities, endogenous ecotin of wild-type bacteria provides complete protection against the lectin pathway-related and the complement-unrelated attack, and partial protection against the alternative pathway-related damage. In all, ecotin emerges as a potent, versatile self-defense tool that blocks multiple antimicrobial activities of the serum. These findings suggest that ecotin might be a relevant antimicrobial drug target.

## Author summary

Bloodstream infections are major cause of morbidity and mortality in many countries around the globe. As the number of multi-drug resistant pathogenic strains is growing, it is urgent to identify their virulence factors and unveil the corresponding mechanisms of action that enable the pathogen to avoid potent immune response. A microbial inhibitor of serine proteases, ecotin was previously implicated in protecting various pathogenic bacteria and eukaryotic Leishmania species against the host immune system by inhibiting leukocyte elastase. However, the interaction of ecotin with the complement system, which provides a first line defense against pathogens, remained unexplored. We found that ecotin blocks activation of the complement lectin pathway by inhibiting its key activator enzymes, MASP-1 and MASP-2. Furthermore, by inhibiting MASP-3, ecotin also disrupts a fundamental link between the lectin- and the alternative pathways. We provide evidence that *E*. *coli* cells devoid of ecotin are extremely vulnerable to complement-mediated lysis and they are also potently killed by some complement-independent antimicrobial factors of human serum. These findings could explain the observations of other research groups reporting that ecotin is crucial for the survival of pathogenic microbes in the host. Our results therefore also highlight ecotin as a potential target of future antimicrobial therapies.

## Introduction

Ecotin is a canonical serine protease (SP) inhibitor first isolated from *Escherichia coli* [1]. Three unique features provide ecotin with unusually broad specificity, yet high affinity. Ecotin has: i) a “one-size-fits-all” methionine P1 residue [2] acceptable for the S1 pocket of many different SPs; ii) a peculiar binding mechanism whereby the ecotin homodimer “chelates” two SPs, each being tweezed between the primary binding site of one monomer and the secondary binding site of the other one [3,4] and iii) structural plasticity [5] enabling accommodation to a large set of SPs having different binding surfaces. Ecotin inhibits all three major pancreatic SPs, trypsin, chymotrypsin and elastase, and its function was first assumed to protect *E*. *coli* in its natural habitat, the colon [1]. Later, ecotin was shown to inhibit several plasma SPs, such as activated coagulation factor X (fXa) [6] and activated coagulation factor XII (fXIIa), as well as plasma kallikrein [7], but none of these enzymes were considered as physiologic targets.

Since then, ecotin orthologs were found in several microbes including human pathogens, such as *Yersinia pestis*¸ *Pseudomonas aeruginosa* and *Burkholderia pseudomallei* [8,9] and even in eukaryotic organisms such as the pathogenic protozoa Trypanosomatida, including *Leishmania major* [10]. Ecotin orthologs from *E*. *coli*, *Y*. *pestis*, and *P*. *aeruginosa* were shown to inhibit neutrophil elastase (NE) secreted by neutrophils and macrophages during inflammation, and this activity was interpreted as a potential defense mechanism [11].

The complement system (CS) belongs to the humoral arm of the innate immune system and it is among the first defense lines against pathogenic microbes. It can be activated through three pathways, the classical (CP), the lectin (LP) and the alternative pathway (AP) [12]. The activity of all three pathways rely on specific SPs. Key enzymes of the LP are mannan-binding lectin (MBL)-associated serine protease (MASP)-1 and -2 [13], while MASP-3 is responsible for the activation of pro-factor D (pro-FD), the zymogen of the key AP-activating enzyme, factor D (FD) [14]. Activation of the LP and the AP is independent from the slowly developing adaptive immune response, therefore these two pathways can unleash an immediate attack against invading microbes [15]. Accordingly, LP- and AP-inactivating capacity could provide the pathogens with substantial advantage during colonization of the host.

Interestingly, although the CS is a major, SP-dependent antimicrobial defense arm [15,16], ecotin has not been assessed as a CS-inhibitor. We studied the mechanism of action of ecotin in the past [17,18] and are investigating the molecular mechanisms of complement activation in the present [14,19,20]. When the crystal structures of our *in vitro* evolved, monospecific Schistocerca Gregaria Protease Inhibitor-related MASP inhibitors, (SGMI)-1 and SGMI-2 were solved in complex with MASP-1 and MASP-2, respectively [21], it revealed that the SGMIs and ecotin share analogous intramolecular interactions stabilizing the protease binding loop. Therefore, we tested *E*. *coli* ecotin as a LP inhibitor, and this has subsequently lead to the comprehensive study described here. This revealed that recombinant *E*. *coli*, *Y*. *pestis*, *P*. *aeruginosa* and *L*. *major* ecotin orthologs inhibit MASP-1-, MASP-2 and MASP-3 with varying relative potencies, while all four ecotin orthologs block LP-activation in normal human serum (NHS) with high and equal efficiency. We also demonstrated that *E*. *coli* ecotin inhibits LP-activation in mouse and rat sera as well.

Factor D (FD) is the initiator of the AP, and we have recently revealed that MASP-3 is the exclusive activator of pro-FD in resting human blood [14,22]. Here we show that *E*. *coli* ecotin readily blocks MASP-3-driven pro-FD activation. Using wild type *E*. *coli* cells and their derivatives lacking the *eco* gene (ecotin *KO*), we also show that endogenous ecotin protects bacteria against the attack of the LP and the AP, as well as against a complement-independent, heat resistant antimicrobial mechanism of heat-inactivated serum (HIS). All these findings unequivocally demonstrate that after 36 years of its discovery, ecotin emerges as a potent microbial defense factor modulating the immune response of the host by targeting several key proteases of the immune system. Accordingly, ecotin could be considered as a relevant antimicrobial drug target.

## Results

**Fig 1:**
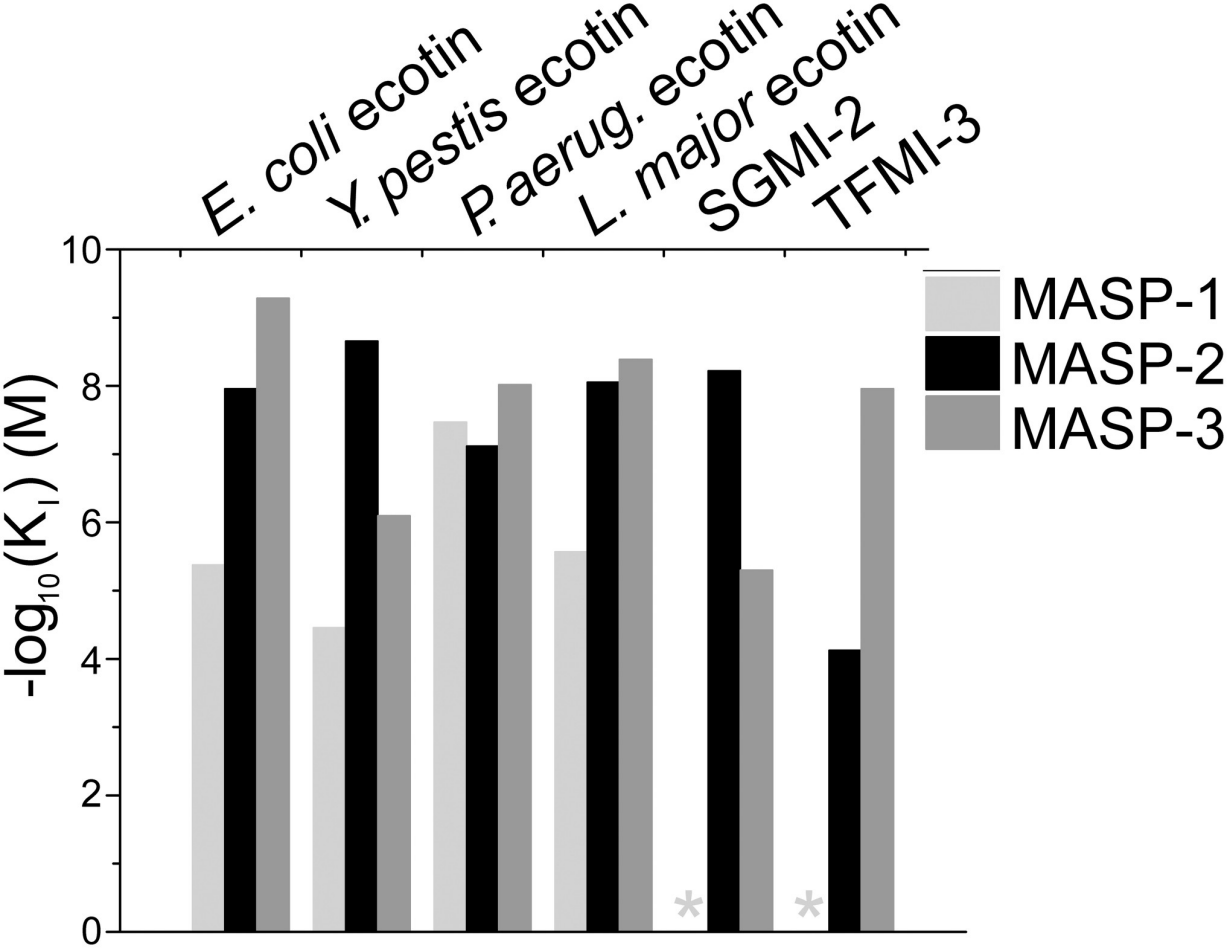
Ecotin orthologs from pathogenic microbes inhibit all three MASP enzymes. Affinities of the four ecotin orthologs as well as SGMI-2 and TFMI-3 toward MASP-1 (light grey bars), MASP-2 (black bars) and MASP-3 (dark grey bars) are shown as–log_10_(*K*_*I*_) (M) values. Asterisks (_*_) indicate that no inhibition could be detected.

**Fig 2:**
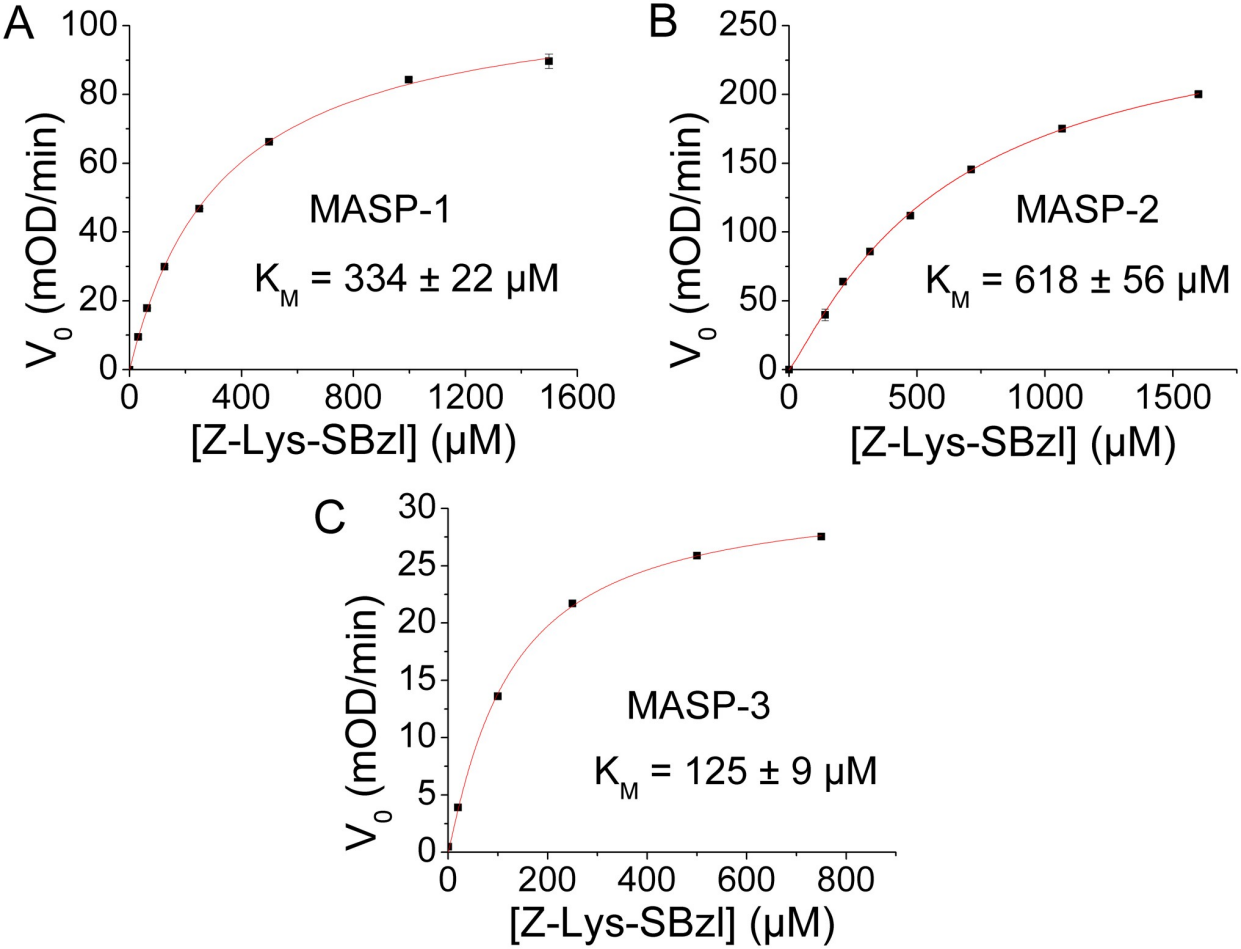
*K*_*M*_ values of Z-L-Lys-SBzl on MASP-1 (A), MASP-2 (B) and MASP-3 (C) were determined. The *K*_*M*_ values of MASP-1, MASP-2 and MASP-3 for Z-L-Lys-SBzl are 334μM, 618μM and 125μM, respectively. Symbols represent the average of three independent measurements. Error bars represent the SEM. The Hill equation was fitted to the data. The *K*_*M*_ values are given in μM ± the standard error.

Binding affinities of the four ecotin orthologs to the catalytic fragments of MASP-1, MASP-2 and MASP-3 have been determined in the form of dissociation constants, which, for reversible inhibitors are referred to as equilibrium inhibition constant (*K*_I_) values. The values shown in Table 1 were measured as explained briefly below. In a series of samples increasing concentration of inhibitor was mixed with a preset concentration of enzyme, and after reaching binding equilibrium, the free enzyme concentration was determined through measuring the residual enzyme activity by adding an appropriate substrate. The obtained dose response curve was fitted to the appropriate kinetic model as explained in the Materials and methods section. The lower the *K*_I_ value the higher the affinity is, and the differences can span orders of magnitudes. Therefore, for easier comparison of the affinities, we also illustrated them as -log_10_ of the *K*_I_ values in Fig 1. Some of the measurements delivered apparent *K*_I_ values. For calculating the corresponding genuine *K*_I_ values, we determined the *K*_M_ values for each MASP enzyme-substrate pair (Fig 2.) (see Materials and methods).

**Table 1:** *K*_I_ values of four ecotin orthologs, SGMI-2 and TFMI-3 towards all three MASP enzymes. The ecotin-MASP affinities in Table 1 were measured at thermodynamic equilibrium as described in the Materials and methods. For tight-binding inhibition assays, enzyme and inhibitor concentrations need to be low, close to the *K*_*I*_ value, and reaching the equilibrium requires several hours-long incubation. Then, small synthetic substrates are added, and their conversion rates report on the concentration of the free enzyme. In these short readout time assays the small substrates compete only with the relatively high-off-rate inhibitors and in this case the measured apparent *K*_*I*_ value needs to be corrected with *K*_*M*_ (see *K*_*M*_ values in Fig 2). On the other hand, small substrates do not compete with the low off-rate tight-binding inhibitors for enzyme binding.

Inhibitor	*KI* (M)		
	MASP-1	MASP-2	MASP-3
*E*. *coli* ecotin	4.2 ± 0.3*10^−6, a^	1.1 ± 0.1*10^−8, a^	5.2 ± 0.3*10^−10^
*Y*. *pestis* ecotin	3.5 ± 0.17*10^−5, a^	2.2 ± 0.1*10^−9, a^	8.0 ± 0.3*10^−7, a^
*P*. *aeruginosa* ecotin	3.4 ± 0.2*10^−8, a^	7.6 ± 0.0*10^−8, a^	9.6 ± 0.8*10^−9, a^
*L*. *major* ecotin (ISP2)	2.7 ± 0.2*10^−6, a^	8.8 ± 0.6*10^−9, a^	4.1 ± 1.0*10^−9^
SGMI-2	N.D.^b^	6.0*10^−9, b^	5.2 ± 0.3*10^−6, c^
TFMI-3	N.D.^d^	7.5 ± 0.3*10^−5, d^	1.1 ± 0.1*10^−8, d^

Data are the average of at least two independent measurements ± SEM. N.D. No inhibition could be detected.

^a^Data was corrected for competition with the synthetic substrate (see details in Materials and methods).

^b^Data from [21].

^c^Data from [13].

^d^Data from [14].

### Ecotin orthologs are from weak to moderate inhibitors of MASP-1

From the four orthologs, *P*. *aeruginosa* ecotin has a 34 nM binding affinity towards MASP-1. On the other hand, *Y*. *pestis*, *L*. *major* and *E*. *coli* ecotin orthologs are weak MASP-1 inhibitors with *K*_I_ values in the 10^−5^–10^−6^ M range (Fig 1, Table 1), the *E*. *coli* inhibitor having a 4.2 μM *K*_I_. In a surface plasmon resonance study, Gaboriaud et al. reported no detectable interaction between *E*. *coli* ecotin and MASP-1 [23]. The highest MASP-1 analyte concentration they applied was below the *K*_I_ determined in this study, therefore there is no conflict between the two observations.

### Ecotin orthologs are potent MASP-2 inhibitors

Ecotin orthologs are potent inhibitors of MASP-2 with *K*_I_ values in the 10^−8^–10^−9^ M range (Fig 1, Table 1). *E*. *coli* ecotin inhibits MASP-2 with a *K*_I_ of 11 nM, which is close to the SPR-based *K*_D_ value of 24.7 nM reported by Gaboriaud et al. [23]. Importantly, most ecotin orthologs are as good, or even better MASP-2 inhibitors than SGMI-2, a monospecific, high-affinity inhibitor of the enzyme we developed previously *via* directed evolution [21].

### Ecotin orthologs are highly potent MASP-3 inhibitors

Except *Y*. *pestis* ecotin, which has a *K*_I_ value of 8.0×10^−7^ M, the other three orthologs are highly potent inhibitors of MASP-3 with *K*_I_ values in the 10^−9^–10^−10^ M range (Fig 1, Table 1). *E*. *coli* ecotin, with its 0.5 nM *K*_I_ value, is a ~20-fold more efficient MASP-3 inhibitor than TFMI-3, our monospecific MASP-3 inhibitor developed by phage display [14]. Previously, no natural MASP-3 inhibitors have been identified. Our findings suggest that by inhibiting MASP-3, ecotin expressing microbes could disrupt the activatory connection between the LP and the AP [14]. The 3–4 orders of magnitude higher inhibitory potency of the tested ecotin orthologs on MASP-2 and MASP-3 versus MASP-1 might reflect the higher evolutionary conservation of MASP-2 and MASP-3, which are present in all vertebrates, while MASP-1 is apparently missing from birds and most fish [24–26].

It is worth noting that, by using SPR, Gaboriaud et al. measured a much weaker, 600 nM affinity between *E*. *coli* ecotin and MASP-3 [23]. An important difference between the two experiments is that they used a single MASP-3 SP domain construct, which lacked even the SP-structure stabilizing activation peptide. In this study, we used a MASP-3 catalytic fragment construct containing two complement control protein (CCP) modules connected to the SP domain through the activation peptide. The observed thousand-fold difference in affinities should correspond to the more stable, native structure of the MASP-3 SP domain in our experiments.

### *E*. *coli* ecotin inhibits MASP-3 through a “slow-binding” mechanism, and blocks MASP-3-driven pro-FD conversion *in vitro*

**Fig 3:**
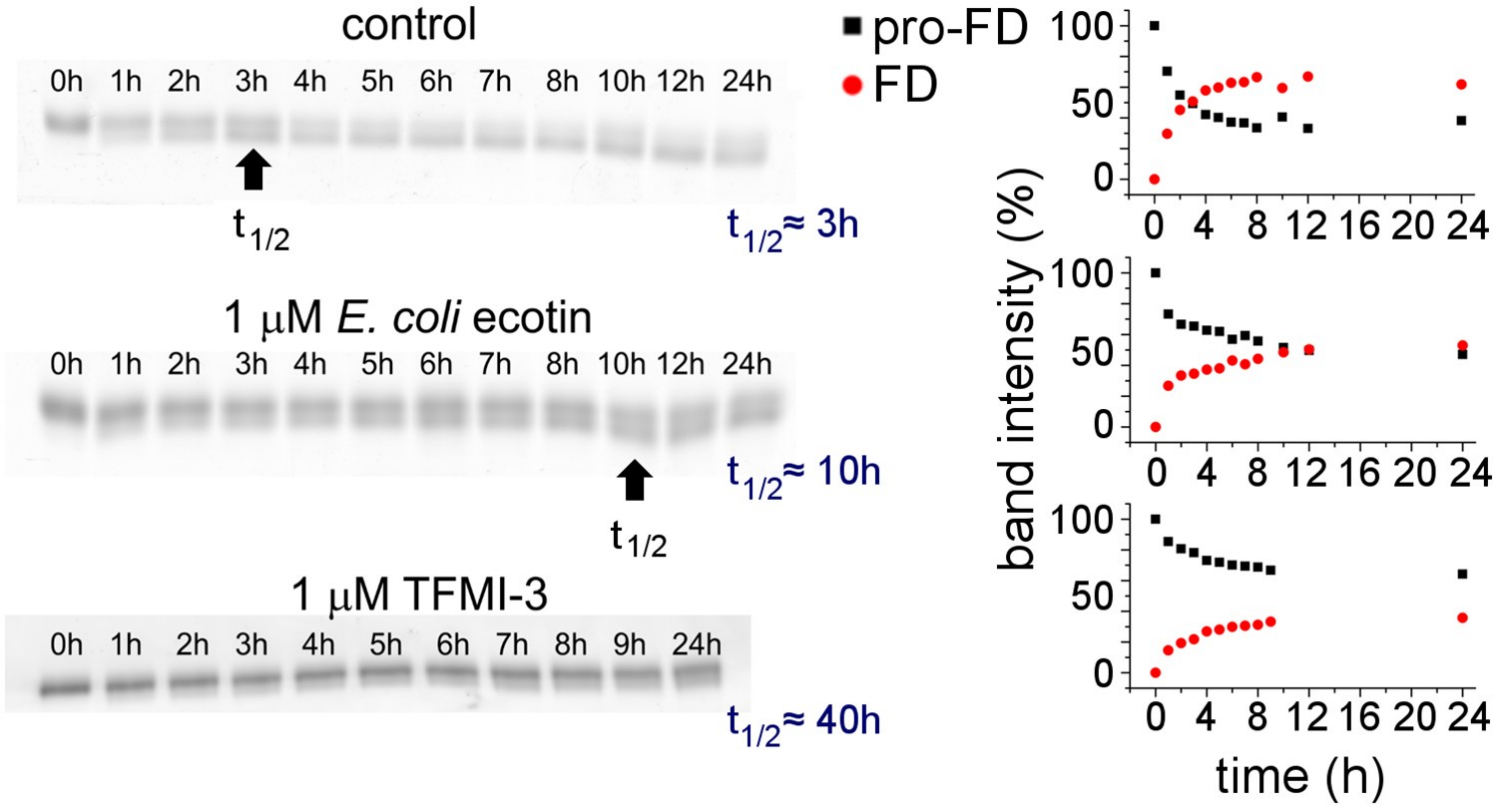
*E*. *coli* ecotin inhibits the activation of pro-FD to FD in NHS. Its efficiency is compared to that of our already published *in vitro* evolved MASP-3 inhibitor, TFMI-3. 1 μM ecotin increases the half-life of fluorescently labeled pro-FD in NHS from 3 hours to 10 hours, while 1 μM TFMI-3 prolongs that to ~40 hours.

**Fig 4:**
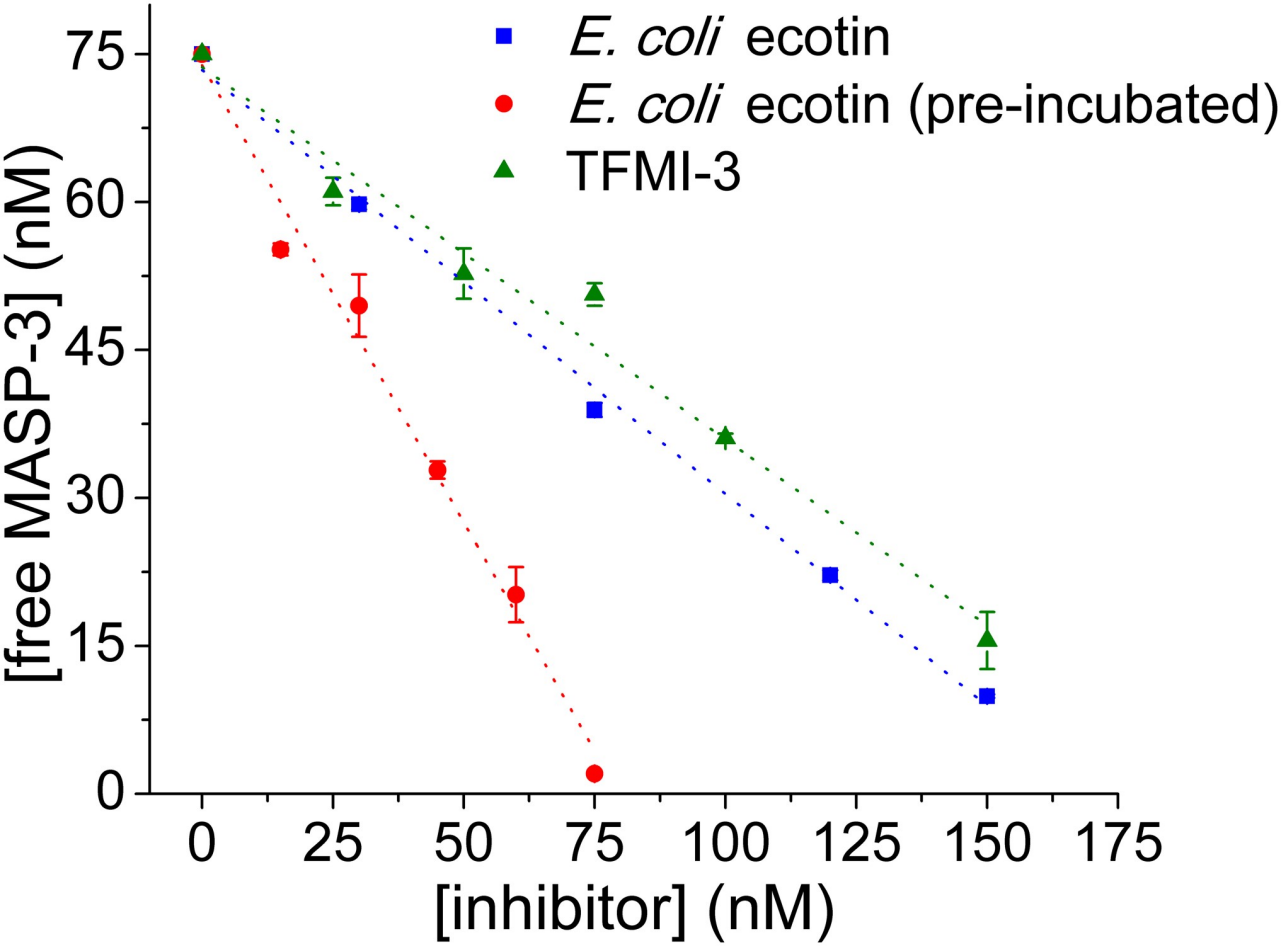
*E*. *coli* ecotin inhibits the MASP-3 driven pro-FD conversion. *E*. *coli* ecotin was shown to be a high-affinity inhibitor of MASP-3 (see Table 1) in a small synthetic substrate-based *in vitro* enzyme-inhibition test, where enzyme and inhibitor reached thermodynamic equilibrium. We measured how efficiently ecotin inhibits MASP-3 under non-equilibrium conditions by competing with pro-FD, the natural substrate of the enzyme. We found that ecotin inhibits MASP-3 driven pro-FD conversion in a concentration-dependent manner (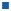), but only slightly more effectively than the 20-fold lower affinity MASP-3 inhibitor TFMI-3 (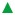) (see Table 1). After pre-incubating ecotin with MASP-3 to approach thermodynamic equilibrium, the interaction of the two proteins became stronger and stoichiometric (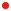) in accordance with the subnanomolar *K*_I_ value (see Table 1). This suggests that the interaction reaches equilibrium slowly. Data points are the average of two independent measurements. Error bars represent the SEM. Dotted lines are used to visually emphasize the trends.

The ecotin-MASP *K*_*I*_ values in Table 1 were measured at thermodynamic equilibrium using small synthetic substrates. These values do not necessarily predict how efficiently ecotin would compete with pro-FD, the large natural substrate of MASP-3, which could bind the enzyme stronger than small substrates. To test this, first we applied a chromatography-based assay using purified recombinant proteins [22] and measured how *E*. *coli* ecotin hinders pro-FD conversion. Low sensitivity of the method required high enzyme and inhibitor concentrations, which facilitate the rapid attainment of equilibrium. We therefore applied short incubation time. While ecotin inhibited the pro-FD conversion in a concentration-dependent manner, it was only slightly more efficient than TFMI-3, which has 20-fold lower affinity (Fig 3). Extending the pre-incubation time between MASP-3 and ecotin significantly increased the binding affinity, which, in agreement with the 0.5 nM equilibrium *K*_I_ value (Table 1), resulted in a stoichiometric linear titration (Fig 4). This incubation time dependence revealed a slow-binding complex formation mechanism. Slow-binding, which is quite common for high-affinity complexes, suggests that the structure of the enzyme and/or the inhibitor needs to go through a slow conformational change during the maturation of the complex.

### *E*. *coli* ecotin inhibits activation of pro-FD in NHS

We also tested how *E*. *coli* ecotin inhibits pro-FD conversion in NHS using an assay format already described [14,22]. Briefly, the assay was developed for in situ detection of the pro-FD converting capacity of plasma or serum samples. As these complex biological samples contain many different proteins including endogenous pro-FD and FD, the assay relies on recombinant, fluorescently labeled pro-FD added to the plasma or serum sample. The kinetics of the conversion was followed by sodium dodecyl sulfate polyacrylamide gel electrophoresis (SDS-PAGE) combined by measuring the fluorescence intensity of the pro-FD and/or FD band. For testing how ecotin can block endogenous pro-FD conversion, the inhibitor was pre-incubated with the serum for 10 minutes before labeled pro-FD was added. In accordance with the previously mentioned slow-binding mechanism, thermodynamic equilibrium between MASP-3 and ecotin could not be reached, and in the first hour, the rate of pro-FD activation was almost unaffected. However, pro-FD activation was almost completely inhibited after ~6 hours indicating formation of the high-affinity equilibrium ecotin-MASP-3 complex. While 1 μM TFMI-3 prolonged the 3 hour serum half-life of pro-FD to ~40 hours [14], 1 μM of the slow-binding *E*. *coli* ecotin prolonged it to 10 hours (Fig 3).

### *E*. *coli* ecotin is a moderate inhibitor of FD and of the AP-type C3 convertase, C3bBb

We tested whether *E*. *coli* ecotin inhibits FD and/or C3bBb, the two activator proteases of the AP. FD-inhibition was measured in a simple photometric enzyme-inhibition assay using the small molecule synthetic substrate, Z-Lys-SBzl (see Materials and methods), and the corresponding *K*_I_ value is 4.06 μM.

**Fig 5:**
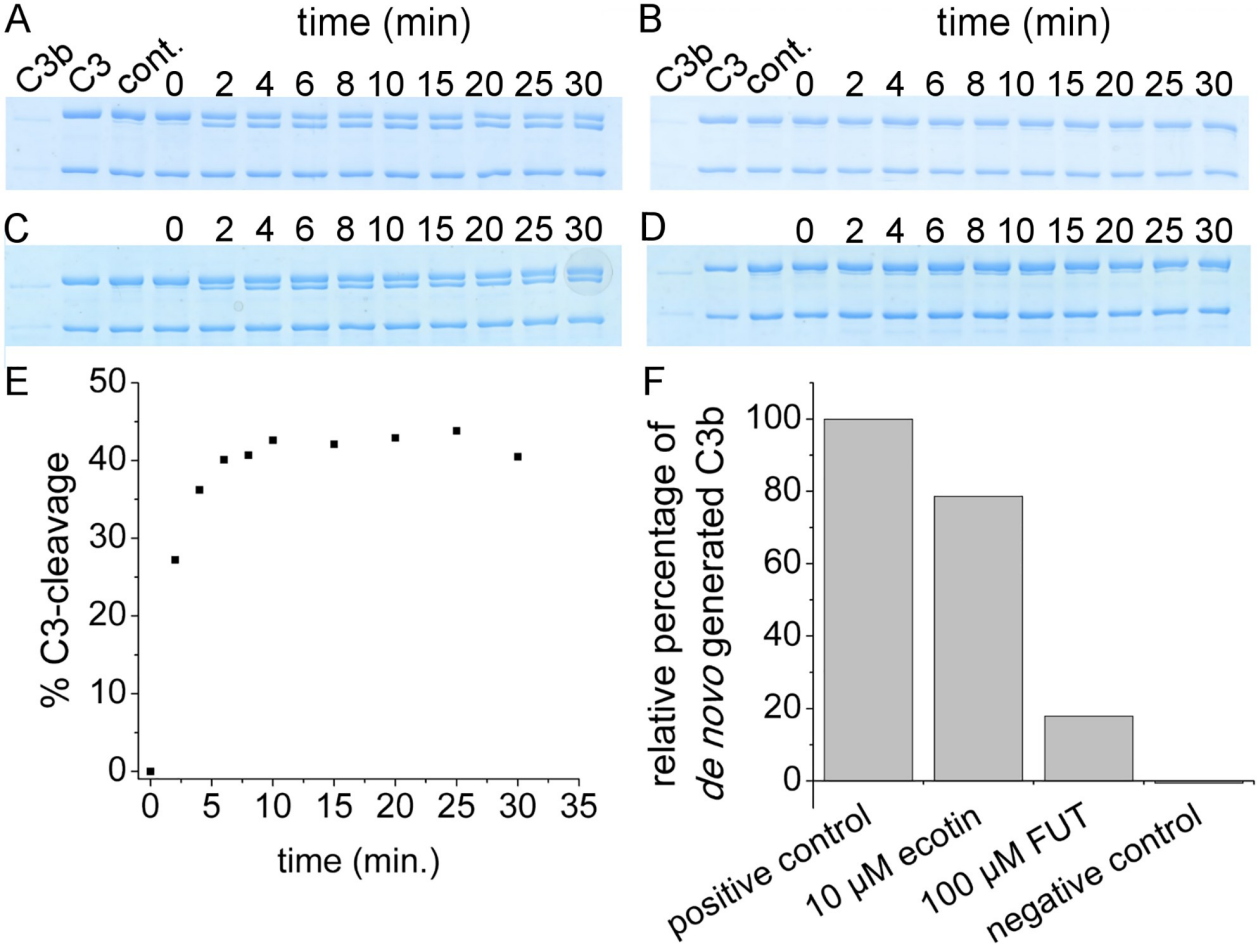
*E*. *coli* ecotin inhibits FD-driven C3bBb-formation and C3bBb-mediated C3b-production. Approximately 45% of C3 was cleaved when no inhibitor was present (positive control) (A). When no FB and FD were present, no C3-cleavage occurred (negative control) (B). In (C) the sample contained 10 μM *E*. *coli* ecotin, while in (D) 100 μM FUT. As the AP-type C3bBb C3-convertase has a short half-life, no C3-clevage occurs after 10 minutes (E), hence, we compared the amounts of C3b generated in 10 minutes after adding FD (F). At 10 μM, *E*. *coli* ecotin decreased the production of C3b by 20% (C, F), while at 100 μM, FUT, the small-molecule general complement-inhibitor, decreased C3b-production by 80% (D, F).

Combined inhibitory activity of ecotin on FD and C3bBb was tested in an SDS-PAGE based densitometry-analyzed C3-cleavage assay, and it was compared to that of the small molecule pan-specific complement inhibitor, FUT-175. In this assay, isolated FB and C3b serve as components for the formation of the pro-convertase, C3bB, isolated C3 serves as substrate, and C3bBb formation and C3-clevage is initiated by adding isolated FD to the mixture containing no inhibitor (positive control), 10 μM ecotin, or 100 μM FUT. In the positive control approximately 45% of the C3 was cleaved to C3b and C3a in the first 10 minutes (Fig 5A and 5E), after which no further cleavage occurred due to the short half-life of C3bBb. Therefore, we quantified and compared the C3b-contents of the 10-minute samples (Fig 5F). At 10 μM concentration *E*. *coli* ecotin reduced C3b-formation by 20% (Fig 5C and 5F), while 100 μM FUT-175 exerted 80% inhibition (Fig 5D and 5F).

### *E*. *coli* ecotin is a weak inhibitor of C1s while it is inactive on C1r

By using catalytic fragments of C1s and C1r and their appropriate synthetic substrates, we tested how ecotin inhibits these key enzymes of the CP. We could not detect any inhibition of C1r, while ecotin inhibited C1s with a 25.15±4.45 μM *K*_*I*_ value representing a very weak inhibition. These findings are in agreement with previous SPR-based results of Gaboriaud et al. [23] who immobilized ecotin on the chip and by injecting up to 2 μM C1s or C1r catalytic fragments, did not detect any interaction.

### Effects of ecotin orthologs on the three complement pathways in NHS

We measured the inhibitory potency of ecotin variants on the three individual CS pathways using NHS.

**Fig 6:**
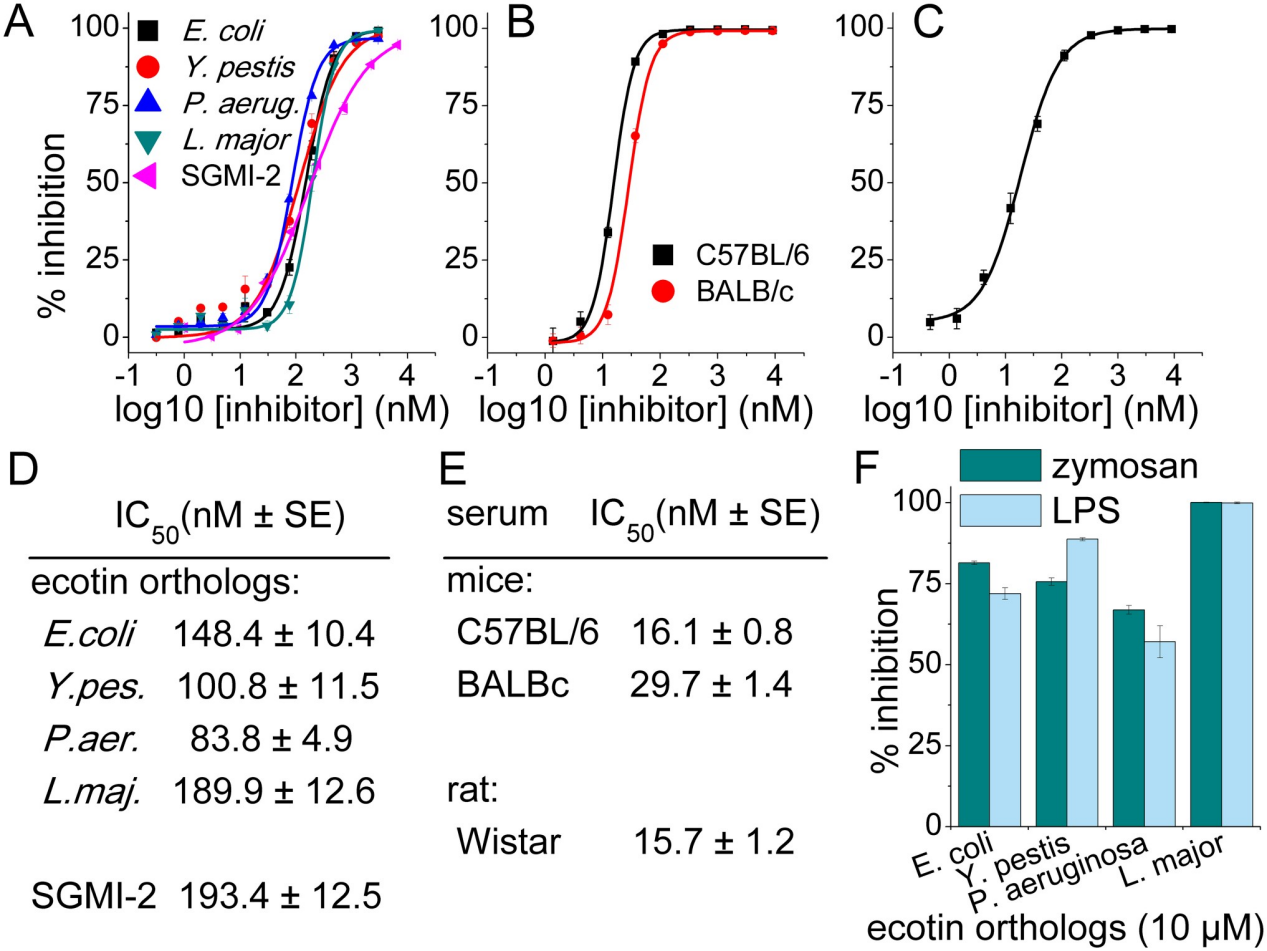
Ecotin is shown to inhibit LP- and AP-activation in human, and LP-activation in mouse and rat serum. C3-deposition on mannan-coated plates from dilute sera was measured as a function of inhibitor-concentration. All four tested ecotin orthologs are equally potent inhibitors of LP-activation in 50-fold diluted NHS. Their effects are comparable to that of our previously evolved selective MASP-2 inhibitor, SGMI-2. (A) *E*. *coli* ecotin inhibits the LP in analogous tests using 60-fold diluted pooled sera of C57BL/6 and BALB/c mice. (B), and 60-fold diluted serum of Wistar rat (C). IC_50_ values for human and rodent serum are listed in (D) and (E), respectively. In the about 10-fold more concentrate, 6-fold diluted NHS used for the alternative pathway inhibition tests, 10 μM of the ecotin orthologs provide ortholog-specific, but significant inhibition, *L*. *major* ecotin being the most potent one (F). Symbols represent the average of at least two measurements. Error bars represent the SEM.

In accordance with their MASP inhibition profile, ecotin orthologs readily blocked mannan-triggered LP-driven C3-deposition in 2% NHS (Fig 6A). Our second-generation *in vitro* evolved MASP-2 inhibitor, SGMI-2 [13,21] was used as an internal reference to assess relative LP-inhibitory efficiencies of the inhibitors. The IC_50_ value range of the ecotin orthologs is 84–190 nM, the highest value being essentially identical with that of SGMI-2 (194 nM) (Fig 6D). Overall, this is in accordance with the *K*_I_ values of the inhibitors towards MASP-2. While the IC_50_ range is narrow, it is notable, that the most efficient LP inhibitor is *P*. *aeruginosa* ecotin, which has the best-balanced MASP inhibitory profile. While the other three orthologs are stronger MASP-2 inhibitors, they are significantly weaker MASP-1 inhibitors. Apparently, inhibitors having moderate affinities toward both MASP-1 and MASP-2, can be similarly or more efficient LP inhibitors than more specific, high-affinity MASP-2 inhibitors. We observed the same phenomenon for our first-generation sunflower trypsin inhibitor (SFTI) related MASP inhibitor (SFMI) compounds [27]. All ecotin orthologs provided complete LP-inhibition in the low micromolar range.

LPS- or zymosan-triggered, AP-driven C3-deposition was measured in 16.7% (6-fold diluted) NHS representing a serum concentration about 8-fold higher than the one used for the CP- and LP-activation assays. All ecotin orthologs exerted potent AP-inhibition in this test, but required a higher, 10 μM inhibitor concentration (Fig 6F). This result is in agreement with the above mentioned synthetic substrate based and SDS-PAGE based observations, where ecotin was shown to be a micromolar FD-inhibitor, and a moderate inhibitor of *de novo* C3bBb production as well as of C3bBb-driven C3b production (Fig 5F).

**Fig 7:**
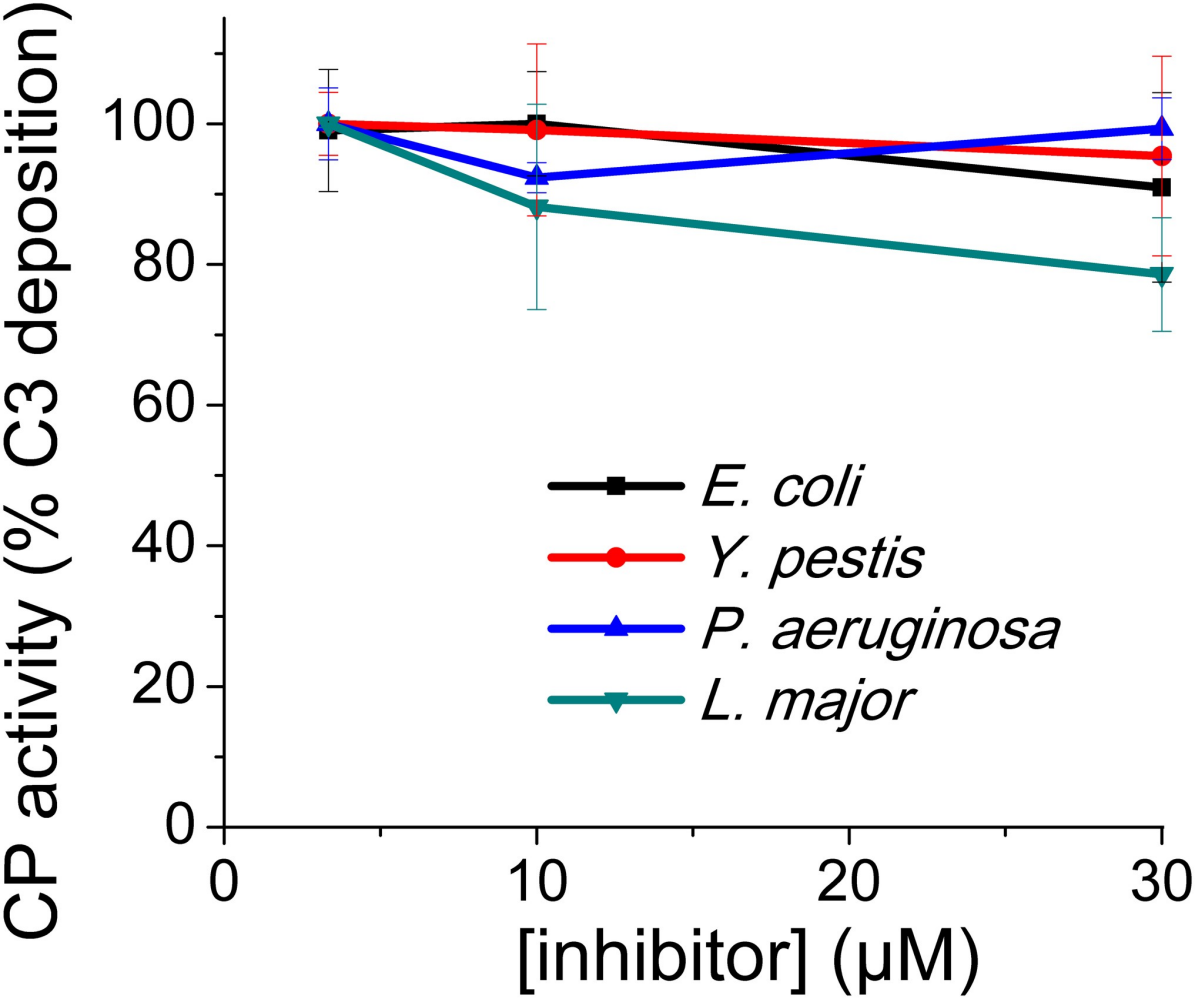
Ecotin orthologs do not inhibit the classical pathway (CP). CP was triggered from 50-fold diluted NHS on IgG coated plates. Symbols represent the average of four parallel measurements. Error bars represent the SD.

As we showed that *E*. *coli* ecotin is a very weak C1s inhibitor, which does not inhibit C1r at all, we already anticipated that this ecotin ortholog will not inhibit the CP. This was verified and we also found that none of the ecotin orthologs inhibited IgG-triggered CP-driven C3-deposition in 2% NHS (Fig 7). This indicates that the other ecotin orthologs are also ineffective against C1s and C1r.

### Ecotin orthologs do not inhibit the C4b2a-type C3 convertase

Potent LP-inhibitory activity of all tested ecotin orthologs demonstrated that ecotin remains functional in NHS i.e. it is not degraded by any serum protease. As the CP- and the LP-inhibitory ELISA test conditions differ only in the immobilized target, all ecotin orthologs must be present in the CP-assay in functional form. Lack of CP-blocking activity therefore clearly demonstrates that ecotin orthologs are inactive against the C4b2a-type C3 convertase.

### *E*. *coli* ecotin is a potent LP inhibitor in rat and mouse serum as well

To test whether ecotin can block mannan-triggered LP-activation in other mammalian species as well, we used pooled sera of C57BL/6 and BALB/c mice and an individual serum sample of a Wistar rat, and tested the inhibitory effect of *E*. *coli* ecotin in C3-deposition ELISA tests. *E*. *coli* ecotin proved to be effective LP inhibitor in all three tests with IC_50_ values of 16 nM, 30 nM and 16 nM in C57BL/6 or BALB/c mice and Wistar rat, respectively (Fig 6B, 6C and 6E). The results reveal that the physiologic and pathologic roles of LP inhibitory activity of ecotin can be readily tested in these animal models. Moreover, cross-species activity of ecotin suggests an ancient role in microbial defense that could manifest in broad phylogenic groups of hosts.

### Endogenous ecotin protects bacteria in NHS against C3-deposition, membrane attack complex (MAC) formation and MAC-mediated cell-killing

**Fig 8:**
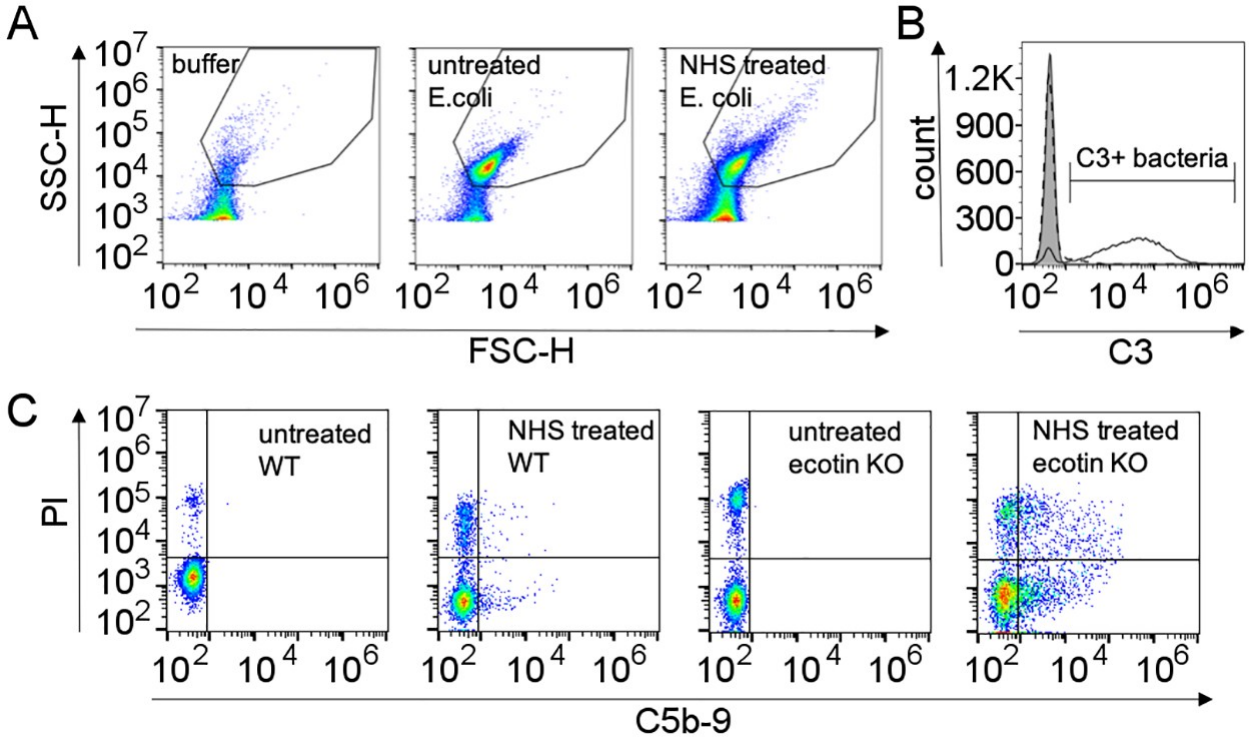
Gating strategy for analyzing C3 and C5b9 deposition by flow cytometry. Thresholds and gating on bacteria population on FSC-SSC plots was set up and verified by analyzing signals of 0.2 μm filtered buffer. Even the filtered buffers contained low amount of particles in the size range of bacteria (A) causing low level of inhomogeneity in the bacterial gate. A representative C3 staining of 2% NHS treated ATCC 23505 cells (filled grey histogram untreated cells, dashed line wild type cells, solid black line ecotin *KO* cells) is shown on panel B. Fluorescence intensity was measured on 20,000 cells captured inside the bacteria gate and C3+ marker was set on the negative control untreated sample. Because of the biasing effect of the few non-bacterial particles in the bacteria gate, the statistical analysis (MFI) of C3-deposition was conducted on the cells under the C3+ marker. Control samples had no event in this region (B). C5b9/PI positive cells were determined by double staining of the cells after treatment. 20,000 cells in the bacteria gate were analyzed, quadrants were set on the untreated, labeled samples. Sample preparation alone induces some cell death (PI positivity) in the samples. Percentage of C5b-9/PI positive cells inside bacteria gate (upper right quadrant) was used for data analysis (C).

We tested how endogenous ecotin protects bacteria against complement-mediated attack of NHS by comparing wild type and ecotin *KO* variants of two *E*. *coli* strains (ATCC 23505 and ATCC 12014) having different lipopolysaccharide (LPS) surfaces. Briefly, wild type or ecotin *KO* bacteria were incubated in 1–16% NHS for 30 min and deposition of activated C3 fragments and C5b-9 were measured by flow cytometry. Gating strategy is shown in a representative case in Fig 8. Cell death was also monitored by propidium iodide (PI) staining and proliferation-based viability tests.

The O-antigen of the ATCC 23505 strain is a mannan homopolymer [28], which activates the complement LP [29]. On the other hand, the more typical O-antigen (O55) of the smooth LPS of ATCC 12014 is composed of repeating pentasaccharide units lacking mannan groups but containing several acetylated carbohydrate components [30].

**Fig 9:**
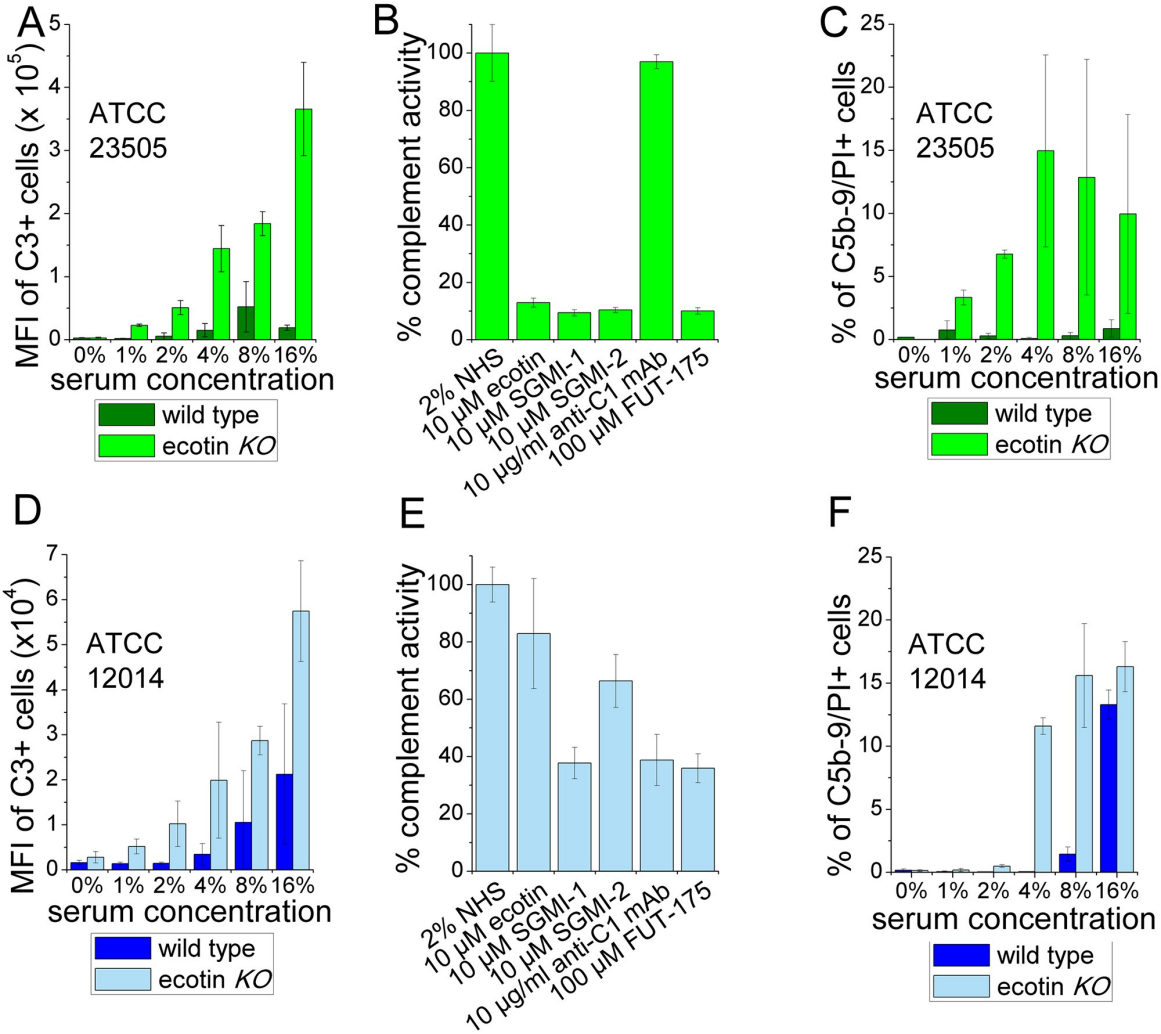
Endogenous ecotin protects *E*. *coli* against C3 and C5b-9-deposition from NHS. The extent and the initiating pathway of CS activation were studied on two wild type and ecotin *KO* coli strains, the polymannan LPS carrying ATCC 23505 and the smooth LPS containing ATCC 12014. Cells were treated with increasing concentrations of NHS and labeled for deposited C3 fragments, and C5b-9 as described in Materials and methods. Mean fluorescence intensity (MFI) values of C3 positive cells (A, D) and percentage of C5b-9/PI positive cells inside the bacteria FSC-SSC gate were determined and is shown (C, F). Cells were treated with 2% NHS containing the indicated inhibitors, and the MFI of deposited C3 was determined as for panel A and D. MFI of deposited C3 without inhibitor was considered 100% complement activity (B, E). Data represent median +/- SD of three independent experiments. MFI of the C3-positive and C5b-9/PI positive ATCC 23505 ecotin *KO* cells was orders of magnitude higher than that of the wild type cells (A, C). This strain should trigger the LP, and indeed, SGMI-1 and SGMI-2, inhibiting MASP-1 and MASP-2, respectively, as well as exogenously added ecotin provided complete inhibition, while the anti-C1q mAb had no effect. The broad-specificity protease-inhibitor, FUT-175 was included as a control (B). Similar trends were found with the ATCC 12014 cells. This strain is less intensely attacked by the complement, and while it is also protected by its endogenous ecotin, the ratio of C3 MFI values and percentage of C5b-9/PI positive cells are smaller (D, F). All exogenously added inhibitors decreased C3-deposition on ATCC 12014 ecotin *KO* cells, but SGMI-1 and anti-C1q mAb were the most efficient. Therefore, this strain was attacked by all three, but dominantly by the classical and the alternative pathway (E).

**Fig 10:**
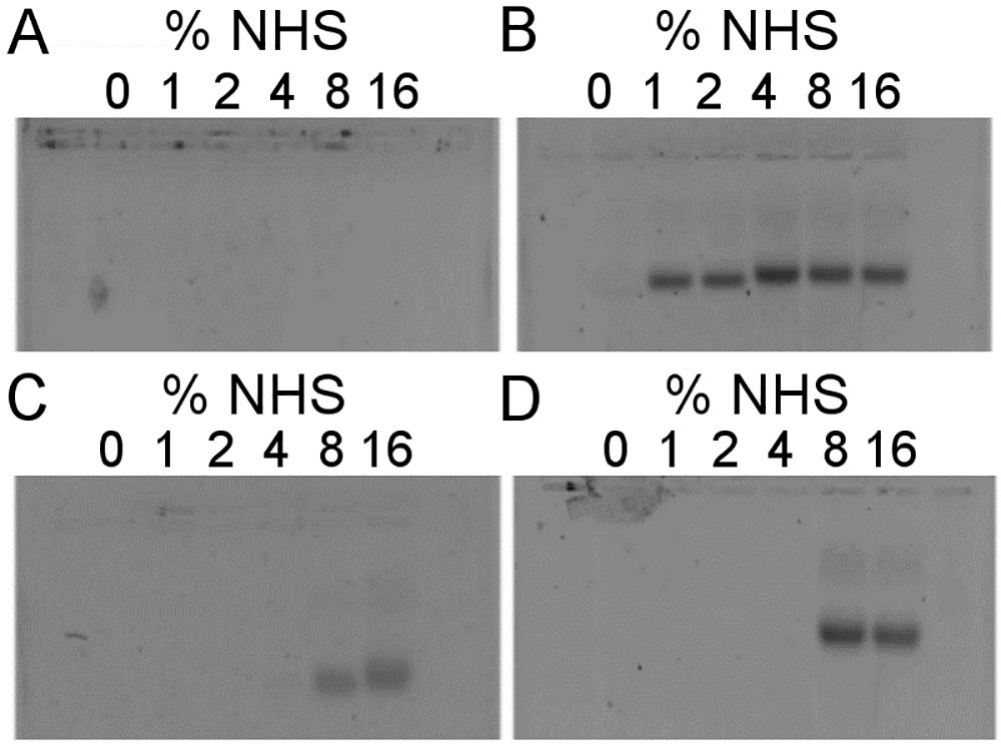
Intense NHS-driven cell-lysis is detected by DNA-content of cell supernatants. We examined MAC formation both by C5b-9 specific antibodies and by propidium iodide (PI) to identify bacteria killed by MAC-driven cell lysis. In several samples, where cells were treated with above 4% NHS, the C5b-9 MFI values of the C5b-9/PI positive cells topped or even decreased (Fig 9C and 9F) indicating some artefacts. Here we show that the supernatants of these cells contain large amounts of DNA suggesting that the highest-labeled C5b-9/PI positive population has been disintegrated. Supernatants obtained during the preparation for flow cytometry experiments were applied onto agarose gel and DNA was detected with SYBR Green. No significant amounts of DNA leaked from wild type ATCC 23505 cells upon incubation with serum up to 16% NHS (A), while its ecotin *KO* derivative already lysed in as low as 1% NHS (B). The supernatant of ATCC 12014 cells contain detectable (C), while that of its ecotin *KO* derivative significant amount (D) of DNA upon treatment with 8% or 16% NHS. The % values indicate the NHS concentration the cells were treated with.

C3 and C5b-9-deposition was significantly higher on the ecotin *KO* versions of the two strains in terms of the mean fluorescence intensity (MFI) values of the C3 positive population and the ratio of C5b-9/PI positive cells (Fig 9A, 9C, 9D and 9F). The percentage of C3 positive cells was also higher in the ecotin *KO* versions of the two strains at lower serum concentrations. At the highest NHS concentration (16%) the percentage of C3 positive cells reached a plateau in both the wild type and ecotin *KO* samples. In further experiments and analyses the C3 MFI values of the C3 positive cells were determined and compared. In above 4% NHS, the lysis of the ecotin *KO* cells was so intense, that large amount of DNA was detected in the cell supernatant (Fig 10), and the ratio of the C5b-9/PI positive cells topped or even decreased (Fig 9C and 9F) indicating that the highest-labeled population disintegrated. In contrast, on the wild type cells, only low level of C3b and C5b-9 was detected up to 8% NHS for the ATCC 12014 strain and up to the highest tested NHS concentration of 16% for the ATCC 23505 strain suggesting a highly efficient protective role of ecotin enabling only a sub-lethal level of MAC formation (Fig 9C and 9F). In fact, this low level MAC formation could be of utmost importance as it could open an exit route for the periplasmic ecotin through the outer membrane to reach the site of the surface-localized complement attack. The observed difference in the two wild type strains is apparently due to the different relative contributions of the three CS pathway to the attack, as explained below.

To assess which CS pathways are in action against the ecotin *KO* cells, we used exogenously added selective inhibitors: SGMI-2, which blocks only the LP [13], SGMI-1, which blocks both the LP and, when applied at higher concentrations, the LPS-triggered AP [13,19] and an anti-C1q monoclonal antibody (mAb) (MW1828, Sanquin), which blocks the CP. Ecotin was also applied exogenously as a control to test whether it provides comparable protection to that of the endogenous periplasmic inhibitor present in the wild type bacteria. We conducted these tests both in the context of the above mentioned C3-deposition assay as well as with the cell proliferation assays described below.

Briefly, in the proliferation-based cell-killing assay, constant amount of mid-log phase cells was treated for 1h with diluted serum either lacking or containing exogenously added inhibitors. The bacteria were then transferred into large excess of broth and the optical density (λ = 600 nm) was measured after 5h incubation.

**Fig 11:**
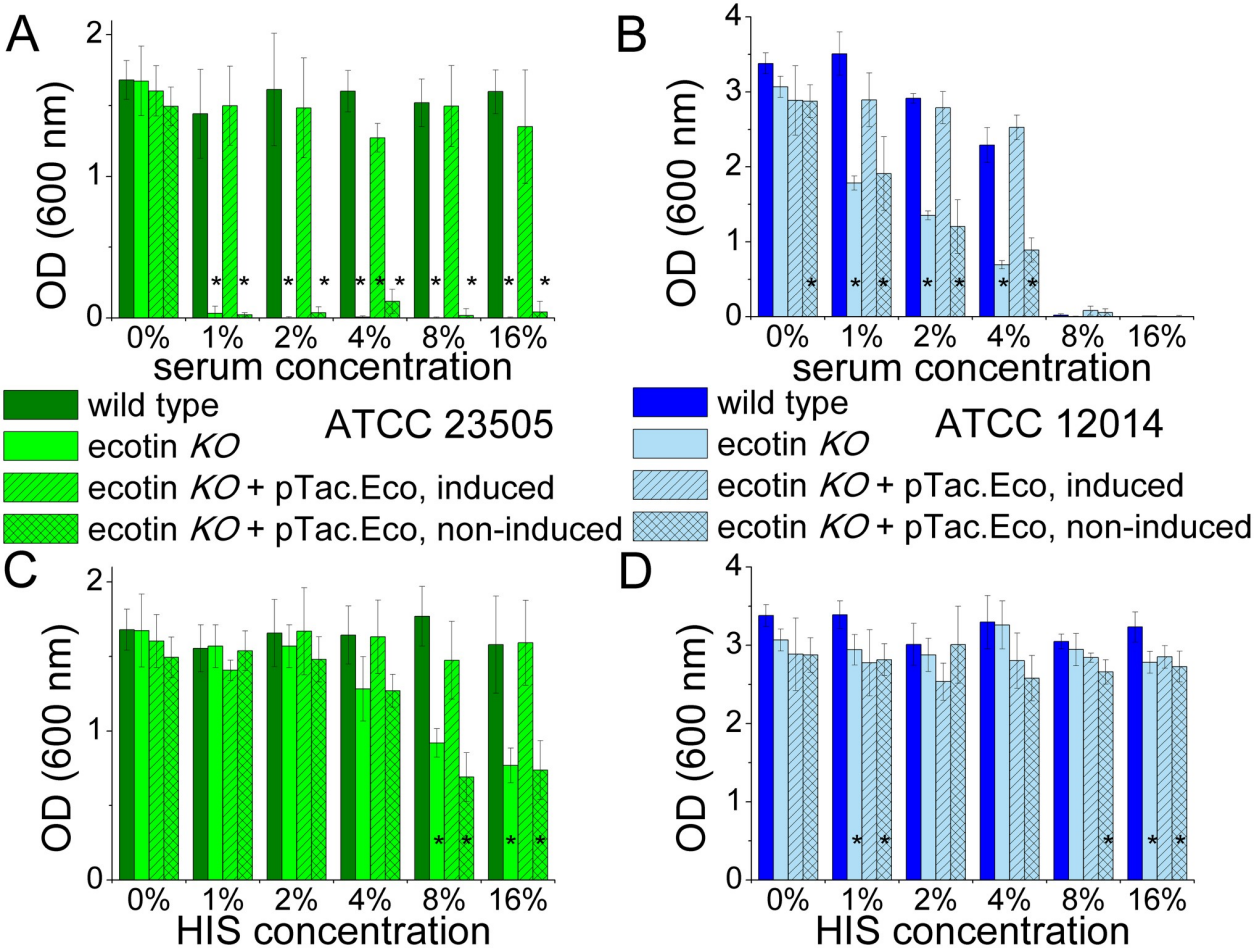
Endogenous ecotin protects bacteria against bactericidal effects of serum. Wild type cells of both strains were more resistant to the bactericidal effects of serum than their ecotin *KO* versions. Accordingly, when the ecotin *KO* cells were provided with the ecotin gene using an IPTG-inducible vector, non-induced cells behaved as the untransformed, original ecotin *KO* cells, while 50 μM IPTG-induction restored the resistant, wild type phenotype in these cells as follows. ATCC 23505 ecotin *KO* cells and their transformed but non-induced version were killed even by 1% NHS, while wild type cells and transformed, IPTG-induced ecotin *KO* cells were completely protected against 16% NHS (A). Up to 4% NHS, endogenous ecotin provided significant protection for the wild type and the transformed, IPTG-induced ecotin *KO* ATCC 12014 cells, but at higher NHS concentration, both the ecotin *KO* cells, regardless of IPTG-induction, and the wild type cells died (B). We also tested if heat inactivated serum (HIS) affects viability of these strains. Interestingly, above 4% concentration, HIS eliminated about 50% of the untransformed ATCC 23505 ecotin *KO* cells and their transformed but non-induced derivative, while the original wild type and the transformed and IPTG-induced ecotin *KO* version were completely protected (C). Wild type and ecotin *KO* ATCC 12014 cells were practically resistant to HIS, regardless of transformation and IPTG-induction (D). Results are from three parallel experiments. Error bars represent the SD, asterisks (*) represent p < 0.05 (two-tailed Student’s t-test) between the OD values of wild type and ecotin *KO* cells and their derivatives.

In agreement with the C5b-9-deposition assay, the ecotin *KO* ATCC 23505 strain was efficiently attacked and killed by the serum, as almost no viable cell remained after an exposure to as low as 1% NHS. On the other hand, endogenous ecotin of the wild type ATCC 23505 strain provided perfect protection in a broad range of serum dilution up to 16% (Fig 11A).

We tested whether increased serum-susceptibility of the ecotin *KO* ATCC 23505 and ATCC 12014 cells is due only to the lack of endogenous ecotin. To do so, we transformed these cells with an isopropyl β-D-1-thiogalactopyranoside (IPTG)-inducible vector to restore periplasmic ecotin expression. Constructing the expression vector and measuring periplasmic ecotin levels of the cells is described in the Materials and methods. Briefly, ecotin levels were measured through chymotrypsin inhibitory activity of the isolated periplasmic fraction as follows: 20 nM final concentration of bovine chymotrypsin was mixed with serial dilutions of periplasmic fractions and the dilution corresponding to 50% enzyme inhibition was determined. We verified that the periplasm of ecotin *KO* cells does not contain ecotin. Isolated periplasmic fraction of wild type cells readily inhibited chymotrypsin. The periplasm of transformed but non-induced cells showed hardly detectable chymotrypsin inhibitory activity. Ecotin levels in the periplasmic fraction of transformed and IPTG-induced ATCC 12014 ecotin *KO* cells exceeded the wild type level 2.6-fold, while the corresponding ratio for the ATCC 23505 ecotin *KO* cells was 1.5-fold. Therefore, periplasmic ecotin level was restored or slightly increased over the wild type level in both strains.

Most importantly, in the ATCC 23505 ecotin *KO* strains, endogenously expressed ecotin perfectly restored wild type phenotype. Without induction, the vector-carrying ecotin *KO* cells were as susceptible to cell-killing as the untransformed ecotin *KO* cell. In contrast, IPTG-induced ecotin expression rescued these cells providing them with a level of protection practically identical with that of the wild type (Fig 11A).

**Fig 12:**
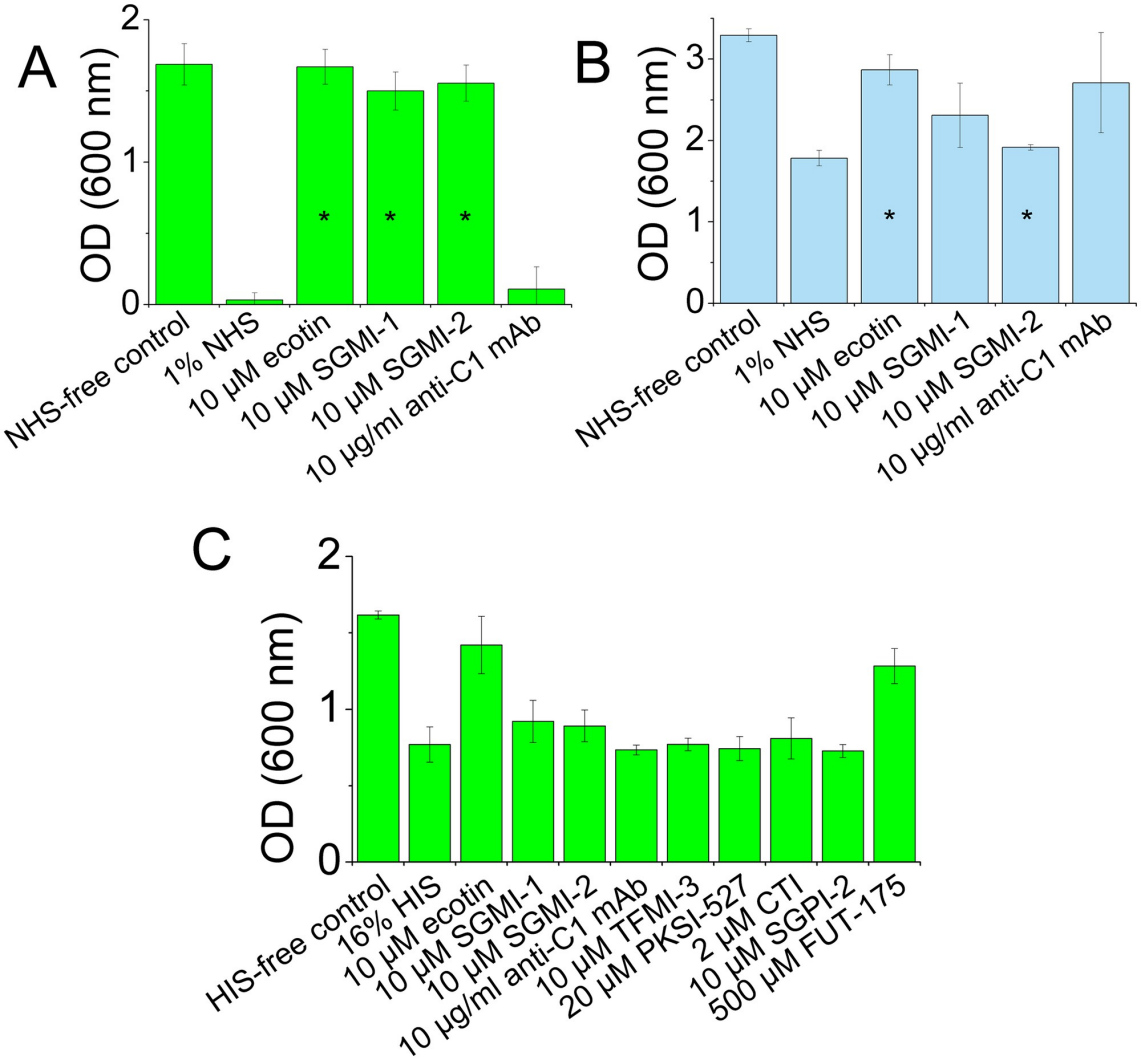
Mechanism of the bactericidal effects investigated by exogenous inhibitors. Just like for C3-deposition in Fig 9, we tested the efficacy of exogenously added inhibitors also on the bactericidal effect. We used 1% NHS where the observed bactericidal effect of heat-stable factors (see Fig 11C) is negligible. Exogenously added SGMI-1 and -2, as well as ecotin rescued the ATCC 23505 ecotin *KO* strain, while anti-C1q mAb exerted minimal effect (A) supporting the dominant role of LP-activation. ATCC 12014 ecotin *KO* cells were rescued by the anti-C1q mAb, ecotin and SGMI-1, while SGMI-2 exerted a minor effect suggesting that all three complement pathways are in action, but the classical and alternative pathways dominate (B). We also attempted to identify the factors responsible for complement-independent bactericidal effects of 16% HIS. Only ecotin and the general serine protease inhibitor FUT-175 could diminish this attack (C). Data are from at least two parallel measurements, asterisks (*) representing p < 0.05 (two-tailed Student’s t-test) when comparing the OD values of samples treated with inhibitors to those of 1% NHS. As data in (C) were obtained from two parallels, no statistical significance was calculated. Error bars represent the SD.

The flow cytometry experiments above demonstrated that the ATCC 23505 bacteria were attacked only by the LP, as both SGMI-1 and SGMI-2 could completely block C3-deposition, while the anti-C1q mAb provided no protection (Fig 9B). Exogenous ecotin also provided full protection in agreement with the fact that wild-type ATCC 23505 cells and the rescued *KO* cells were completely protected by their endogenous ecotin (Figs 9B and 11A). The cell proliferation experiments yielded the same results: all LP-inhibitory compounds provided full protection for the cells, while the anti-C1q mAb had no activity (Fig 12A).

Likewise, the flow cytometry measurements above showed that the ATCC 12014 bacteria were also attacked by the complement system, but, in terms of deposited C3, at a tenfold lower level (Fig 9D), and through different mechanisms (Fig 9E). Anti-C1q mAb and SGMI-1 provided almost complete protection against C3-deposition approaching the level achieved by the broad-specificity protease inhibitor FUT-175 [31] (344960, Sigma-Aldrich), while SGMI-2 had much lower protecting effects (Fig 9E) demonstrating that LP-activation on this strain is negligible. On the other hand, high efficiency of the anti-C1q mAb treatment identified an essential role of CP activation, and the observed drop in C3-deposition can be interpreted as the cancelled contribution of the CP, plus the CP-triggered positive feedback loop provided by the AP, the latter one generating up to 90% of the deposited C3b [32]. High efficiency of SGMI-1 (Fig 9E) cannot be explained by LP-inhibition, as we already concluded that LP was hardly triggered. However, it is in agreement with our previous finding that at the high concentration applied here, SGMI-1 is a strong inhibitor of LPS-triggered AP activity [19]. In all, high efficiency of SGMI-1 suggests that it inhibits both spontaneous AP-activation as well as the CP-triggered AP feedback loop.

The cell proliferation tests provided coherent results: endogenous ecotin of the wild type ATCC 12014 strain, that does not inhibit CP-activation, provided significant, but only partial protection for the bacteria (Fig 11B), most probably by partial inhibition of the AP amplification loop. When SGMI-1 or the anti-C1q antibody was added exogenously to the ecotin *KO* cells, these provided significantly stronger protection than SGMI-2, which had only a marginal effect (Fig 12B).

Again, importantly, in the ATCC 12014 ecotin *KO* strains, endogenously expressed ecotin perfectly restored wild type phenotype in the cell-killing assay. Without induction, the vector-carrying ecotin *KO* cells were as susceptible to cell-killing as the untransformed ecotin *KO* cell, while ecotin expression induction rescued these cells providing them with a level of protection practically identical with that of the wild type cells (Fig 11B).

### Endogenous ecotin protects bacteria against a complement-independent, heat-stable antimicrobial effector mechanism of serum

As the activity of the CS is heat sensitive, heat-inactivated serum (HIS) was used in the flow cytometry and cell proliferation assays as a typical negative control serving as a baseline to deconvolute complement-specific effects. In the flow cytometry assays, as expected, no C3-deposition was detected from HIS. Yet, based on the cell proliferation assays, and to our great surprise, the polymannan O9 LPS-carrying ecotin *KO* ATCC 23505 strain was attacked by HIS in a serum concentration dependent manner (Fig 11C). Lack of deposited C3 fragments verifies that this attack is complement-unrelated. Bactericidal activity of HIS has already been examined by others [33], but the nature of such effects remained enigmatic. The observed bactericidal effect could be ameliorated *via* supplementing the HIS with ecotin or with the broad-specificity protease inhibitor FUT-175 [31]. Extraneous ecotin therefore ablated susceptibility of the cells to a bactericidal effect of HIS. However, our monospecific MASP-inhibitors, SGMI-1, SGMI-2 and TFMI-3 were all ineffective, as were the plasma kallikrein inhibitor PKSI-527 [34] and the FXIIa-blocking corn trypsin inhibitor [35] (Fig 12C). This inhibitor panel demonstrates that the complement-unrelated attack is protease-dependent, but is not driven by the contact system or by any moonlighting activity of the MASP enzymes.

We tested if the observed susceptibility of the ecotin *KO* ATCC 23505 cells to HIS concentration above 4% was due exclusively to the lack of endogenous ecotin, as explained for the other cell-killing tests. We found that in the ATCC 23505 ecotin *KO* strains, endogenously expressed ecotin perfectly restored wild type phenotype. Without induction, the vector-carrying ecotin *KO* cells were as susceptible to cell-killing as the untransformed ecotin *KO* cell. In contrast, IPTG-induced ecotin expression rescued these cells providing them with the same level of protection observed for the wild type (Fig 11C).

## Discussion

Microbial pathogens evolved a variety of complement evasion strategies. These mainly rely on the acquisition or expression of complement regulators and inhibitors, or on the degradation of complement components [36–38], demonstrating that complement inhibition is a crucial defense mechanism of pathogenic microbes. In this paper, we unequivocally prove that ecotin inhibits both antibody-independent CS pathways, the LP and the AP, and it also blocks an apparently complement unrelated antibacterial serum activity. These discoveries place previous findings of other research groups about ecotin as a potential virulence factor into completely new context.

**Fig 13:**
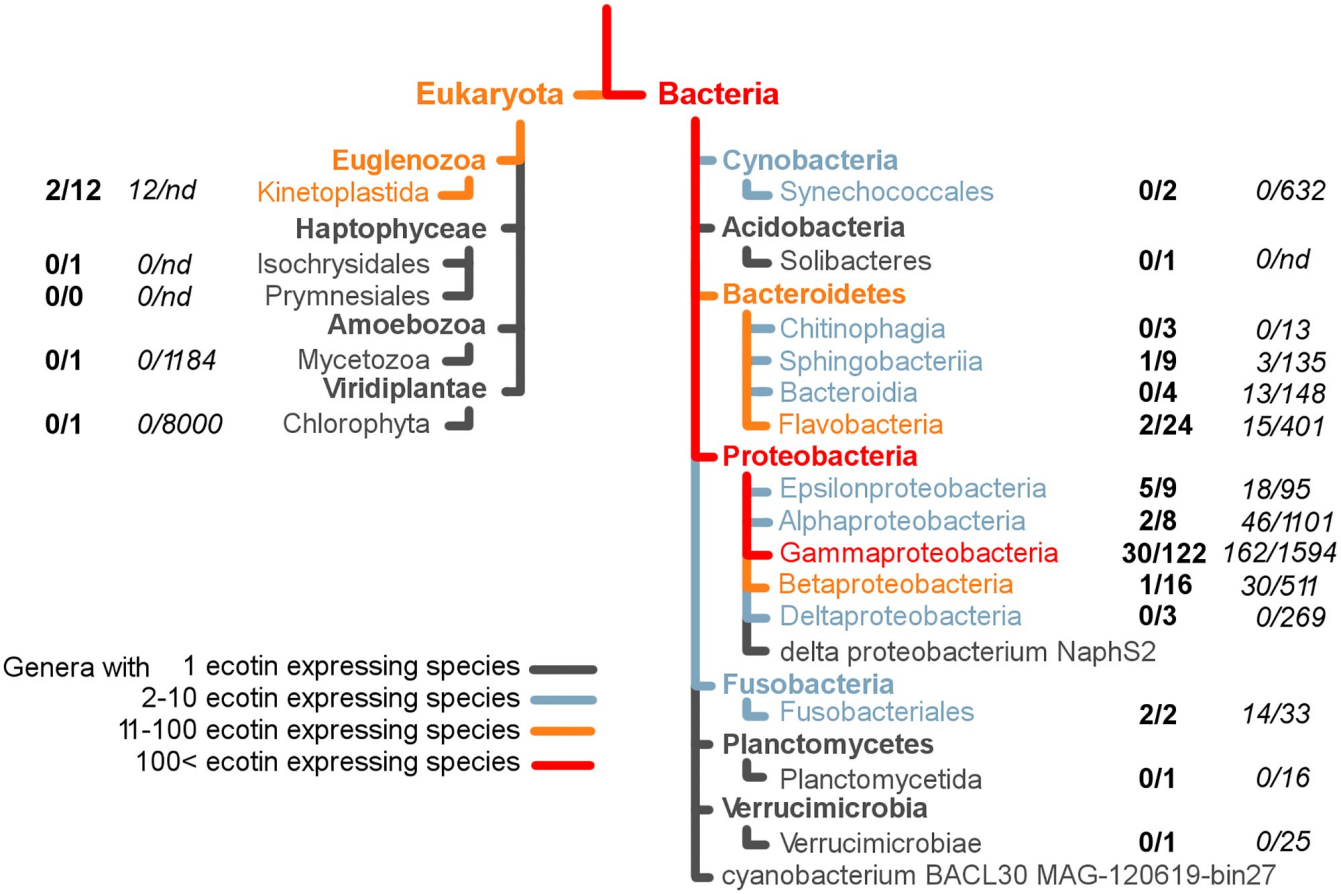
Occurrence of ecotin orthologs in the kingdoms of *Eukaryota* and *Bacteria*. Orthologs of ecotin are present in both *Eukaryota* and *Bacteria* kingdoms. More than three hundred species express at least one ecotin homolog. Among eukaryotic species, the *Trypanosomatidae*, belonging to the *Kinetoplastida* class, contain several species that are causative agents of serious and widespread diseases (based on publicly available pathogen databases (see Materials and methods)), and 12 species of this genus express ecotin homologs (based on the PFAM database https://pfam.xfam.org). Among *Bacteria* two classes are exceptionally rich in ecotin expressing species: *Bacteroidetes* (40 species) and *Proteobacteria* (158 species). Numbers in bold indicate how many pathogens exist among the ecotin expressing species in that genera; while those in italic indicate how many pathogens are among all species in that genera. Figure was made based on PFAM (https://pfam.xfam.org).

Ecotin was discovered 36 years ago in *E*. *coli*. By now, ecotin orthologs have been identified in more than 200 species, and over 40 of these are well-documented opportunistic or obligatory pathogens including *Trypanosoma cruzi*, *Leishmania major*, *Burkholderia pseudomallei*, *Yersinia pestis* and *Pseudomonas aeruginosa* (summarized in Fig 13 and S1 Table). These updated numbers support the decade-old finding that ecotin genes are more frequent in pathogenic species than in non-pathogenic ones [39]. Ecotin producing organisms are widespread among Bacteria, especially within the Gammaproteobacteria subdivision, which contains more than a hundred species harboring ecotin orthologs. In this subdivision, the Enterobacteriaceae family is particularly rich in known pathogenic species expressing ecotin. Moreover, several members of the eukaryotic *Euglenozoa* genus including the human parasites *Trypanosoma brucei* and *Leishmania major* also express ecotin orthologs (S1 Table).

Ecotin has been identified as virulence factor for several pathogenic Gram negative bacteria such as *Yersinia* and *Burkholderia* species and two species of the eukaryotic unicellular pathogen, *Leishmania* as well [8–10,40].

The gastrointestinal pathogen, *Yersinia pseudotuberculosis* is a close relative of the etiologic agent of the bubonic and pneumonic plague, *Yersinia pestis*. When wild type and ecotin *KO Yersinia pseudotuberculosis* cells were injected into the bloodstream of mice in 1:1 ratio, after 5 days, the wild type cells were present in about 30-fold excess compared to the ecotin *KO* cells. It was hypothesized that the *in vivo* function of ecotin might be protection from neutrophil-dependent killing probably by inhibiting neutrophil elastase [8]. *Pseudomonas aeruginosa* is an opportunistic pathogen causing chronic lung infections. Its ecotin was found to be sequestered by the exopolysaccharide, PsI on the bacterial surface and again, it was shown to protect the cells against neutrophil elastase-mediated elimination of the bacteria [41]. This phenomenon is in agreement with the findings of others, who found, that ecotin expression is upregulated in *E*. *coli* and *P*. *aeruginosa* biofilms [42,43]. Since both pathogens form biofilms in certain diseases [44], this might be a mechanism by which ecotin expressing bacteria increase their protection against antimicrobial effectors of the host. For *Burkholderia pseudomallei*, the causative agent of melioidosis, ecotin was shown to be important for intracellular survival of the bacteria in murine macrophages, most probably by inhibiting several proteases of the early endosome and allowing for an escape of the intracellular pathogen into the cytosol. Importantly, compared to the wild type strain, intraperitoneal infection with the ecotin *KO* strain leads to increased time to death and greater survival rate of the infected mice [9].

Ecotin orthologs are present in several species of the eukaryotic parasite *Trypanosomatidae* family including *Trypanosoma cruzi* causing Chagas disease, *Leishmania major* causing zoonotic cutaneous leishmaniasis and *Leishmania donovani* the etiologic agent of visceral leishmaniasis or kala-azar. While it was shown that *Trypanosoma cruzi* is attacked by all three complement pathways, and several parasite proteins were found to attenuate this attack, ecotin was not identified as a complement inhibitor, but was suggested to protect the cells against neutrophil elastase and cathepsin G [45,46].

Similarly, all but one publications on *Leishmania* ecotin orthologs indicated that the main function of these proteins is protection against intestinal proteases in the gut of the insect vector, or against contact pathway-mediated bactericidal effects of the host [47,48], or down-regulation of inflammatory monocytes and monocyte-derived cells [49]. We found only a single, very recent paper reporting that *Leishmania donovani* ecotin ortholog LnISP2 is not only a neutrophil elastase inhibitor, but also a MASP-2 inhibitor [40]. Notably, however, in that study, recombinant LnISP2 was 50-fold less efficient in providing complete MASP-2 / LP-inhibition, than our recombinant *Leishmania major* ISP2 ortholog in analogous tests.

In all, we demonstrated that ecotin inhibits i) LP-activation, ii) LPS / zymosan-triggered AP-activation, iii) MASP-3-based pro-FD activation and iv) an apparently complement-unrelated antimicrobial effect of HIS. All these functions of ecotin, as we showed, provide a proliferative advantage for the ecotin harboring cells.

While the molecular mechanism of LP-inhibition is obvious: ecotin potently inhibits MASP-2, the source of the observed heat-stable bactericidal activity, and thereby the protecting mechanism of ecotin needs to be identified, and the mechanism of ecotin-based AP-inhibition also requires further investigation.

We showed that *E*. *coli* ecotin is a 4 μM FD-inhibitor and that at 10 μM concentration it could provide up to 75% inhibition of AP-activation triggered by LPS- and zymosan surfaces. Moreover, in a system containing purified components, we also showed that, at a lower degree, ecotin blocked FD-driven C3bBb generation and C3bBb-driven C3b generation, demonstrating that *E*. *coli* ecotin is a weak inhibitor of FD, C3bBb, or both. Note, that in the serum tests, ecotin was pre-incubated with the serum, so it could reach binding equilibrium with the active FD contents of the serum. In the solution assay no such pre-equilibrium with FD was possible as the reaction was initiated by FD. Moreover, it is important to note that the above AP-activation systems are not readily comparable. LPS and zymosan-triggered AP-activation are complex, serum-based assays engaging many complement regulatory proteins. Moreover, AP-activation in these tests occur on surfaces, and require a *de novo* C3b-generation phase offering a level of inhibitory intervention missing from the solution phase AP-convertase generating system containing an initial C3b pool provided as purified protein. Previously we showed that MASP-1 is essential for LPS-triggered, but not for zymosan-triggered AP-activation [19]. As *E*. *coli* ecotin is a micromolar MASP-1 inhibitor, this activity might contribute to the inhibition of LPS-triggered but not to zymosan-triggered AP-activation. As long as the exact molecular mechanisms of AP-activation on these surfaces remain unknown, it cannot be excluded that zymosan-triggered AP-activation also relies on a yet to be identified protease, inhibited by ecotin.

MASP-3 was for long considered as a protease lacking any natural serpin-type or canonical inhibitor. Accordingly, the most outstanding property of ecotin is its very high inhibitory potency on MASP-3. As we have recently showed, in resting blood, not perturbed by coagulation or inflammatory processes, MASP-3 is the exclusive activator of pro-FD [14]. As FD is the AP-initiator enzyme, this MASP-3 function is fundamental, and the outstanding MASP-3-inhibitory capacity of ecotin suggests an important biologic function. Note, however, that this ecotin function could not manifest in our AP tests, as in those assays FD was already present in the serum in activated form, or was provided as activated, isolated protein.

As mentioned, in the blood, due to the presence of activated MASP-3, FD circulates mostly in activated form. While systemic control of FD-activation in the blood through complete MASP-3 inhibition should be possible, it would require the continuous presence of large excess of ecotin, which is an unlikely situation in microbial infections. On the other hand, inhibition of MASP-3-driven pro-FD activation can be relevant at the much more common locations of primary infections, which also represent the only niche of normal microbiota, the periphery, including the mucosae. Several opportunistic pathogens, such as *E*. *coli* and *P*. *aeruginosa* colonize at first the mucosae, therefore the earliest encounter between the complement and the bacteria occurs in this environment. The composition of the complement, the concentration of its components, and therefore the mechanism of complement activation might be different in the extravascular environments from those in the blood [50–52]. For example, the level of activated FD at the periphery might be limited by the level of its activators, and if so, ecotin could provide a major defense mechanism against the AP. A similar difference between blood and extravascular environments might prevail for the levels of the LP components. Based on this, we suggest that the potent protective effects of ecotin we demonstrated in dilute serum against the LP, the AP and a complement-unrelated attack imply similar efficient protections in mucosal, interstitial and other extravascular environments.

In all, the activities of ecotin revealed here, together with its previously established neutrophil and macrophage controlling actions demonstrate, that ecotin blocks or attenuates a plethora of powerful antimicrobial attacks of the host innate immune system. These versatile innate immunity-attenuating activities of ecotin might have been evolved in the normal host microbiota to provide general protection for the microbes in the mucosa and other extravascular niches as long as they do not provoke specific IgG-response unleashing (among others) the ecotin-resistant classical complement pathway. The very same ecotin functions, when combined with other virulence factors, should provide important advantages for pathogens as well.

We believe that all previous studies conducted on the interactions between ecotin-harboring microbes and their hosts should be revisited and reevaluated in the light of these new findings. We suggest that ecotin should be put in the focal point of dedicated fundamental research, and as it can be a potential drug target, the new findings might open novel therapeutic options as well.

## Materials and methods

### Ethics statement

All studies using human serum were conducted in strict conformity with the WMA Declaration of Helsinki. Experimental protocols were approved by the local ethics committee (permission number: TUKEB 9190-1/2017/EKU). Informed written consent was obtained for the isolation of peripheral venous blood from the donors.

All procedures using mouse serum were carried out in strict accordance with the recommendations in the Guide for the Care and Use of Laboratory Animals of the National Institutes of Health. The protocol was approved by the Institutional Animal Care and Use Committee at the Icahn School of Medicine at Mount Sinai Center for Comparative Medicine (registration number: D16-00069).

All procedures using rat serum were performed in strict accordance with guidelines set by the National Institutes of Health (USA) and the Hungarian law on animal care and protection. The protocol was first approved by the Institutional Ethical Committee for Animal Care and Use of Semmelweis University and then approved by the Government Office of Pest County Directorate of Food Chain Safety and Animal Health (registration number: PEI-001/3948-6/2014).

### Cell lines and recombinant proteins

Ecotin *KO* variants of *E*. *coli* BL21 (DE3) Star (Invitrogen), ATCC 12014 and ATCC 23505 were made according to [53]. Recombinant ecotin variants were expressed in *E*. *coli* BL21 (DE3) Star (ecotin *KO*) and purified to homogeneity. Recombinant enzymes were expressed and purified according to [54,55]. SGMI-1, SGMI-2 and TFMI-3 were expressed and purified as described in [14,21,56]. The *K*_*I*_ values of ecotin variants against MASP-1 and MASP-2 were determined on synthetic substrates. The *K*_*M*_ values of MASP-1 and MASP-2 for Z-Lys-SBzl were determined (Fig 2). Complement activity was measured in C3-deposition ELISA assays in 2% NHS as described in [13,27]. Similar tests were also conducted with mouse and rat serum as well.

### Reagents

All common laboratory reagents were from Sigma-Aldrich and Merck. High-binding microtiter plates were from Greiner Bio-One.

### Construction of ecotin knockout E. coli strains

The ecotin gene of *E*. *coli* BL21 (DE3) Star (Invitrogen), ATCC 12014 and ATCC 23505 (American Type Culture Collection, ATCC) was replaced with the chloramphenicol acetyltransferase gene (EC 2.3.1.28) using the recombineering plasmid pKD46-RecA as described [53,57]. The plasmid was electroporated into the bacteria, recombinant clones were isolated and the loss of the heat-sensitive plasmid was verified. Successful replacement at the targeted genomic locus was confirmed by PCR, restriction analysis and DNA sequencing using specific primers. The chloramphenicol resistance gene was kept to serve as a stable selection marker for the strains BL21 (DE3) Star ecotin *KO*, ATCC 12014 ecotin *KO* and ATCC 23505 ecotin *KO*. No ecotin could be detected in the periplasm of the recombinant (ecotin *KO*) clones.

### Construction of ecotin expression vectors

The gene coding for the mature *E*. *coli* ecotin sequence (UniProt: P23827) had originally been cloned into the pT7-7 expression vector [17]. Genes coding for the mature ecotin protein sequences of *Y*. *pestis* (UniProt: A4TNJ2), *P*. *aeruginosa* (UniProt: B7UZW2), and *L*. *major* (corresponding to the ISP2 ecotin ortholog) (UniProt: Q4QFD4) were codon-optimized for expression in *E*. *coli*. Cleavage sites of frequently used restriction endonucleases were removed with silent mutations.

The four ecotin genes were obtained as a single synthetic construct (Life Technologies) in which each variant was flanked by cleavage sites of two different restriction endonucleases. The plasmid was digested with the appropriate enzymes in separate reactions. The genes were isolated and amplified by PCR using specific primers that also incorporated NcoI and HindIII sites. The genes of *Y*. *pestis* and *P*. *aeruginosa* ecotins were sub-cloned into the pET-25b(+) periplasmic expression vector (Novagen) with NcoI and HindIII restriction endonucleases. The *L*. *major* ecotin gene was sub-cloned into a modified pETM-11 cytoplasmic expression vector with NcoI and HindIII restriction endonucleases. The *L*. *major* ecotin was produced with an N-terminal His_6_-tag which could be cleaved off with TEV protease.

### Expression and purification of SGMI-2, TFMI-3, pro-FD and the catalytic fragments of MASP-1, MASP-2, MASP-3, C1s and C1r

SGMI-1, SGMI-2 and TFMI-3 were expressed and purified as described previously [14,21,56]. Active catalytic fragments of MASP-1, MASP-2, MASP-3, C1s and C1r containing the CCP1-CCP2-SP domains were expressed and purified as described previously [22,54,55,58,59]. Expression, purification and processing of recombinant human pro-FD was done as described previously [22].

### Expression of ecotin orthologs

*E*. *coli* BL21 (DE3) Star ecotin *KO* cells were transformed with the expression vectors. Transformed cells were grown in LB medium to OD_600 nm_ = 4 at 37°C. Recombinant protein expression was induced with 0.5 mM isopropyl-*β*-D-thiogalacto-pyranoside. After 5 hours, the cell cultures were harvested via centrifugation (4500 g, 4°C, 10 min).

### Purification of *E*. *coli*, *P*. *aeruginosa* and *Y*. *pestis* ecotin orthologs

Cells were resuspended in 100 mL / liters of cell culture 2.5 mM MgCl_2_ and frozen at -20°C overnight. After thawing, the samples were centrifuged (48000 g, 4°C, 20 min) and the supernatants corresponding to the periplasmic fractions were sonicated to disrupt any residual cell debris. Then, 65% (m/v) ammonium-sulphate was added gradually to the sample and the precipitated proteins were collected via centrifugation (48000 g, 4°C, 20 min). The pellet was resuspended in 2.5 mM HCl, and the sample was dialyzed against 2.5 mM HCl overnight at 4°C. The dialyzed sample was centrifuged (48000 g, 4°C, 20 min) and the pH of the ecotin-containing supernatant was set to 7.9 with Tris base. The sample was loaded onto a GE HiTrap Q HP anion-exchange column equilibrated with 10 mM Tris-HCl pH 7.9 buffer. In the case of the *Y*. *pestis* ortholog, the ecotin was the major component of the flow-through. In contrast, the *E*. *coli* and *P*. *aeruginosa* ecotin orthologs were retained by the column and were eluted using a linear NaCl gradient. Fractions containing the ecotin orthologs were further purified with RP-HPLC on a Phenomenex Jupiter 10u C4 300A column equilibrated with water, 0.1% TFA, and eluted with a linear gradient of acetonitrile, 0.09% TFA. The ecotin containing fractions were lyophilized and the proteins were dissolved in water.

### Purification of His_6_-tagged *L*. *major* ecotin (ISP2)

The cells were resuspended in 100 mL per liter of cell culture 50 mM Na-phosphate, 300 mM NaCl pH 7.5 buffer and the suspension was frozen at -20°C. After thawing, the sample was sonicated and centrifuged (48000 g, 4°C, 20 min). The protein was purified on Bio-Rad Profinity IMAC resin equilibrated with 50 mM Na-phosphate, 300 mM NaCl, pH 7.5. The protein was eluted with the same buffer supplemented with 250 mM imidazole and dialyzed against 10 mM Na-phosphate pH 7.5 at room temperature. The His_6_-tag was cleaved off with His_6_-tagged TEV protease. *L*. *major* ecotin was separated from the His_6_-tagged protease and the His_6_-tag by a second Ni^2+^ affinity chromatography. The inhibitor was further purified on a GE HiTrap SP HP cation-exchange column equilibrated with 10 mM Na-phosphate pH 7.5 buffer. The protein was eluted with a linear NaCl gradient.

### Quality control of the inhibitors

Purity of the proteins was confirmed via SDS PAGE. The correct molecular weight of the proteins was verified via mass spectroscopy (ESI-MS).

### Determination of the inhibitor concentration

Concentration of ecotin samples was determined similarly as described elsewhere [11]. Briefly: bovine trypsin (Sigma Aldrich) was dissolved in 10 mM HCl, 10 mM CaCl_2_ and active-site titrated using 4-methylumbelliferyl *p*-guanidinobenzoate [60]. The active concentration of each ecotin stock was determined by titration against the active-site titrated trypsin. Incremental amounts of the inhibitors were incubated with 1 μM trypsin for 10 minutes in 50 mM Tris-HCl pH 8.0, 10 mM CaCl_2_, 0.005% Triton X-100. Residual enzyme activities were measured using 1 mM Nα-Benzoyl-DL-arginine p-nitroanilide. Inhibitor concentration was determined by linear regression analysis.

### Equilibrium binding assays

The equilibrium inhibitory constant (*K*_*I*_) values of ecotin against the catalytic fragments of MASP-1, MASP-2, MASP-3, C1s and C1r as well as against recombinant FD were determined according to the method of Green and Work with the modifications of Empie and Laskowski et al. [61,62]. The measurements were done in 20 mM HEPES, 145 mM NaCl, 5 mM CaCl_2_, 0.05% Triton X-100 pH 7.5 buffer in 200 μl final volume using a BioTek Synergy H4 multimode microplate reader. A fixed concentration of protease was incubated with increasing amounts of the inhibitor in separate samples. Enzyme concentrations were set based on preliminary experiments. After reaching equilibrium, the amount of uninhibited enzyme was quantitated using a thioester substrate combined with an appropriate co-substrate. In most cases, 250 μM Z-L-Lys-SBzl hydrochloride thioester substrate and 500 μM 5,5’-dithiobis-(2-nitrobenzoic acid) (DTNB) as photometric co-substrate were used. For C1s and C1r, 250 μM Z-Gly-Arg-SBzl was combined with 500 μM DTNB. For the tight-binding *E*. *coli* ecotin / MASP-3 interaction the 250 μM Z-L-Lys-SBzl hydrochloride thioester substrate was combined with 40 μM of the high-sensitivity Thiol Fluorescent Probe IV co-substrate. The fluorescence of this co-substrate increases 470-fold upon reacting with thiols [63]. The *K*_*I*_ values were determined from at least two parallel measurements by nonlinear regression analysis in OriginPro software using the following equation:

$$[E]={\left[E\right]}_{0}-\frac{{\left[E\right]}_{0}+{\left[I\right]}_{0}+K_{I}-\sqrt{{\left({\left[E\right]}_{0}+{\left[I\right]}_{0}+K_{I}\right)}^{2}-4{\left[E\right]}_{0}{\left[I\right]}_{0}}}{2},$$

where [E], [E]_0_ and [I]_0_ represent the molar concentration of the free enzyme, the total enzyme, and total inhibitor, respectively.

Based on the literature [7], a *k*_*on*_ of 5.3 x 10^5^ M^-1^ s^-1^ was used to estimate *k*_*off*_ values and subsequently the half-lives of the complexes. For enzyme-inhibitor pairs where the half-life of the complex was comparable with, or shorter than the length of the measurements, only apparent K_I_ values (*K*_I_*) could be determined because there was a genuine competition between the inhibitor and the substrate for enzyme-binding. The *K*_*I*_ values were then calculated by the following equation:

$$K_{I}=\frac{{K_{I}}^{*}}{\left(1+\left[S\right]/K_{M}\right)},$$

where [S] is the molar concentration of the substrate and *K*_*M*_ is the Michaelis constant [64]. For this calculation, the *K*_*M*_ values of the three MASPs for Z-L-Lys-SBzl were determined by us. For FD, a *K*_M_ of 3.79 mM, while for C1s, a *K*_M_ of 190 μM was used, based on the literature [65,66].

### Determination of the K_M_ values

The *K*_M_ values of the three MASPs for Z-L-Lys-SBzl were determined in 20 mM HEPES, 145 mM NaCl, 5 mM CaCl_2_, 0.05% Triton X-100 pH 7.5 buffer in 200 μl final volume using a BioTek Synergy H4 multimode microplate reader. Initial reaction rates (V_0_) were measured at 410 nM for increasing Z-L-Lys-SBzl concentrations in samples containing 500 μM DTNB co-substrate and 10 nM MASP-1, 30 nM MASP-2, or 5 nM MASP-3. Three parallel measurements were done, V_0_ values were averaged and plotted as the function of [S]. The Hill-equation was fitted to the data using the OriginPro software to obtain the *K*_M_ values shown in Fig 2.

### Analysis of the interaction between *E*. *coli* ecotin and MASP-3 using pro-FD as a substrate

These experiments were done as in [22] with the following modifications. The cleavage assays were performed in 50 mM HEPES, 140 mM NaCl, and 0.1 mM EDTA pH 7.4 buffer. For producing a free MASP-3 calibration curve for the subsequent experiments, samples with MASP-3 concentrations in the 0–75 nM range were prepared and supplemented with pro-FD at 10 μM final concentration. The samples were incubated at room temperature for 40 min, diluted 15-fold with ice-cold equilibration buffer (20 mM sodium phosphate, 70 mM NaCl pH 6.8), loaded onto a 4.6 × 50 mm YMC-BioPro SP-F nonporous high-pressure cation exchange column (YMC Europe GmbH), and eluted with a 70–470 mM linear NaCl gradient in the same buffer at 0.8 ml/min. For the analysis of MASP-3 inhibition by *E*. *coli* ecotin or TFMI-3 75 nM MASP-3 and increasing concentrations of the inhibitors were mixed and incubated for 10 min or overnight at room temperature. 10 μM pro-FD (final concentration) was added to the samples. After 40 min the reactions were stopped and the samples were analyzed as described previously. Briefly, AUC values of the pro-FD and FD chromatographic peaks were determined. Free MASP-3 concentrations in the enzyme:inhibitor samples were then calculated based on the above described calibration curve.

### Cy3-labeled pro-FD cleavage in NHS

These experiments were done based on the already described method [22]. A total of 4 μl 1 mg/ml Cy3-labeled pro-FD was added to 40 μl NHS, and the mixtures were incubated at 37°C for 24 hours. Samples were withdrawn at every hour in the first 8 hours, at 10 h, 12 h and then at 24 h on pro-FD conversion. The inhibitors were added to the serum samples at 1 μM final concentration and incubated for 10 minutes prior to addition of the labeled pro-FD. The reaction in the withdrawn samples was immediately stopped by 10-fold dilution with SDS-PAGE sample buffer and heating for 2 min at 95 °C. A total of 2 μl of the cleavage assay samples containing 18 ng Cy3-labeled protein were run on SDS-PAGE under reducing conditions. The gels were scanned with a Typhoon laser scanner (GE Healthcare). Band intensities were quantified by densitometry. Because the two bands were partially overlapping, only the time point at which the intensity of the Cy3–pro-FD band equaled that of the Cy3-FD band could be reliably determined on the gel, and this time point was considered as the half-life of Cy3– pro-FD.

### Testing FD- and C3bBb-inhibiting capacity of ecotin in an *in vitro* AP convertase generating system

An in vitro AP convertase generating system was set up by mixing 100 nM C3b, 30 nM FB and 1 μM C3 that served as substrate for the freshly generated C3bBb-type C3-convertase. This mixture was pre-incubated in 50 mM Tris-HCl, 200 mM NaCl, pH 7.45 buffer at room temperature, for 30 minutes without (positive control) or with inhibitors, before starting AP-convertase generation by adding 10 nM FD. One of the samples contained 10 μM ecotin, while the other contained 100 μM of the pan-specific serine proteinase inhibitor, FUT-175, that inhibits all CS proteases. A sample containing neither FD nor FB served as negative control. From the mixtures that received FD samples were withdrawn at various time points, and were immediately treated with mercaptoethanol and SDS containing gel loading sample buffer at 95 °C. C3b-generation from C3 was assessed by SDS-PAGE. C3 and C3b were applied onto the gel as controls. Extent of C3-cleavage and C3b-generation for the inhibitor containing samples were compared to those in the positive control sample containing no inhibitor and to the samples taken directly after starting the AP-convertase generation by adding FD. This way, we could deconvolute the quantity of C3b added to enable the preliminary C3bB-formation from the C3b generated by the convertase. The results were analyzed *via* ImageJ software.

### Preparation of pooled NHS

Blood was drawn from 10 healthy donors after informed written consent into Vacutainer serum tubes (BD Diagnostics) to produce fresh serum. Blood samples were incubated for 1 h at room temperature to allow complete coagulation. The serum was collected, pooled, and stored at −80 °C until use.

### Preparation of pooled mouse serum

Blood was collected from five C57BL/6 and five BALB/c mice at the age of 6-10 weeks by venipuncture of the facial vein. Blood samples were incubated for 0.5 hours at room temperature to allow coagulation to be completed. The samples were centrifuged at 14,000 rpm for 20 minutes at 4 °C. Serum supernatants were pooled, snap frozen in liquid nitrogen and stored at -80 °C until use.

### Preparation of rat serum

Blood was collected from an individual euthanized Wistar rat. The serum preparation was done as described for human serum preparation.

### Complement ELISA assays

#### Classical pathway (CP) inhibition measurements from NHS

CP activity was measured through detection of C3-deposition as described previously [27], with the following modifications:

1. Greiner high-binding microtiter plates were coated with 10 μg/mL IgG in 15 mM Na_2_CO_3_ pH 9.6, and incubated overnight at 4 °C.
2. Wells were blocked with 1% BSA in TBS pH 7.4 for 1.5 hours at 37 °C, then washed with TBS, 5 mM CaCl_2_, 0.1% Tween 20.
3. Pooled NHS was diluted 50-fold in 10 mM HEPES, 150 mM NaCl, 5 mM CaCl_2_, 5 mM MgCl_2_, 0.1% Tween 20, pH 7.4 (NHS buffer) containing serial dilutions of inhibitors.
4. These mixtures were pre-incubated in single loose tubes (National Scientific Supply Company) for 30 min at room temperature before transferring onto the activator surface.

Polyclonal rabbit anti-human C3c antibody (A0062; DakoCytomation) was used as the primary antibody in 2000-fold dilution and HRP-conjugated anti-rabbit antibody (A1949, Sigma) was used as the secondary antibody in 40,000-fold dilution.

The signal produced by non-inhibited NHS was considered to be 100% activity, while 0% complement activity was defined as the signal produced by 2% NHS treated with 50 μM FUT-175 inhibitor. Results were obtained from four parallel measurements. In all serum tests, the signal developing reaction on the plate was stopped when optical density of the highest signal intensity non-inhibited reaction was between OD 0.5–1.0. The typical background signal was around OD 0.05.

#### Lectin pathway (LP) inhibition measurements from NHS

LP activity was measured through detection of C3-deposition as described previously [27], with the following modifications:

1. Greiner high-binding microtiter plates were coated with 10 μg/mL mannan in 35 mM NaHCO_3_, pH 9.6, and incubated overnight at 4 °C.
2. Wells were blocked with 1% BSA in TBS pH 7.4 for 1.5 hours at 37 °C, then washed with TBS, 5 mM CaCl_2_, 0.1% Tween 20.
3. Pooled NHS was diluted 50-fold in 10 mM HEPES, 150 mM NaCl, 5 mM CaCl_2_, 5 mM MgCl_2_, 0.1% Tween 20, pH 7.4 (NHS buffer) containing serial dilutions of inhibitors.
4. These mixtures were pre-incubated in single loose tubes (National Scientific Supply Company) for 30 min at room temperature before transferring onto the activator surface.

Polyclonal rabbit anti-human C3c antibody (A0062; DakoCytomation) was used as the primary antibody in 2000-fold dilution and HRP-conjugated anti-rabbit antibody (A1949, Sigma) was used as the secondary antibody in 40,000-fold dilution.

The signal produced by non-inhibited NHS was considered to be 100% activity. 0% complement activity was defined as the signal produced by 2% NHS treated with 50 μM FUT-175 inhibitor. Results were obtained from four parallel measurements.

#### LP inhibition measurements from rat serum

In these tests, 60-fold diluted serum was used, otherwise the experiments were performed as described above, because the anti-human C3c polyclonal primary antibody cross-reacts with rat C3c. The signal produced by non-inhibited serum was considered to be 100% activity. 0% complement activity was defined as the signal produced by 60-fold diluted rat serum treated with 50 μM FUT-175 inhibitor. Results were obtained from four parallel measurements.

#### LP inhibition measurements from pooled mouse serum

Nunc MaxiSorp plates were coated with 10 μg/mL mannan in PBS overnight at 4 °C. Wells were blocked with 5 mg/ml BSA in TBS pH 7.4 and washed with TBS, 0.05% Tween-20 (wash buffer) subsequently. Pooled mouse serum was diluted 60-fold in TBS, 5 mM MgCl_2_, 5 mM CaCl_2_, 0.05% Tween-20 containing serial dilutions of *E*. *coli* ecotin. These mixtures were pre-incubated for 15 min at room temperature before transferring 100-100 μl onto the plate. The plates were incubated at 37 °C for 30 minutes and then washed with wash buffer. 100–100 μl HRP-conjugated goat anti-mouse C3 IgG (Cappel #55557) diluted 5000-fold in TBS, 5 mg/ml BSA, 0.05% Tween-20 was applied to the plates and incubated for 1 hour at room temperature. The plates were washed and 100 μl/well TMB substrate (Sigma) was added to the wells. The reaction was stopped by adding 50 μl/well 1 M HCl and the signals were read at 450 nm.

The signal produced by non-inhibited serum was considered to be 100% activity. 0% complement activity was defined as the signal produced by serum treated with 50 μM FUT-175 inhibitor. Results were obtained from three parallel measurements.

#### Alternative pathway inhibition measurements from NHS

Alternative pathway assays were carried out as described for the LP and CP inhibition measurements from NHS, but with the following important modifications:

1. the plates were coated with 100 μg/mL zymosan or with 10 μg/mL LPS (Sigma Aldrich).
2. Modified NHS buffer: 10 mM HEPES, 150 mM NaCl, 5 mM MgCl_2_, 10 mM EGTA and wash buffer: TBS, 0.1% Tween 20, 10 mM EGTA were used to prevent Ca^2+^-dependent activation of the classical and lectin pathways.
3. The final dilution of NHS was 6-fold.

#### Calculation of the half maximal inhibitory concentration (IC_50_) values

IC_50_ values were calculated with using OriginPro software. Data was fitted using the following dose response function:

$$y=A_{min}+\frac{A_{max}-A_{min}}{1+10^{({logIC}_{50}-[I])*p}}$$

where A_min_ and A_max_ represent the minimum and maximum of the fitted curve, respectively, [I] is the inhibitor concentration, p is the Hill slope and y is the degree of C3 deposition.

### Detection of C3-deposition on *E*. *coli* by flow cytometry

Flow cytometry was used to assess C3-deposition on *E*. *coli* cells. *E*. *coli* ATCC 23505, ATCC 23505 ecotin *KO*, ATCC 12014 and ATCC 12014 ecotin *KO* cells were cultured in peptone/beef extract (P/BE) broth, as recommended on www.atcc.org. At OD_600 nm_ = 0.5, cells were chilled on ice, centrifuged and washed twice with 10 mM HEPES, 145 mM NaCl, 5 mM CaCl_2_, 5 mM MgCl_2_ pH 7.4 buffer (NHS-buffer). Cell density was measured, and 3.4 x 10^7^ cells were transferred into 1.5 mL microcentrifuge tubes and pelleted at 15,000 g. The cells were suspended in 200 μL of either NHS or NHS-buffer (negative control) at appropriate dilution, and incubated at 37 °C for 30 minutes. After incubation, cells were washed twice and suspended in 100 μl of 1/500 polyclonal rabbit anti-C3c antibody (A0062, DakoCytomation) or in 100 μL NHS-buffer, mixed thoroughly and incubated at 37 °C for an hour. After washing the cells twice, 100 μl of 1/2000 Alexa Fluor 488 conjugated polyclonal goat anti-rabbit IgG (A21206, Invitrogen) was added to each sample and incubated at room temperature for 30 minutes in dark. Samples were measured on a Beckman Coulter CytoFlex instrument using CytExpert software. SSC threshold values were set to exclude most of debris, and to enable gating on bacterial populations, data from 20,000 cells in the bacteria gate were collected in each sample. All buffers used in flow cytometry samples were filtered (0.22 μm filter), to exclude any contaminating particles in the SSC-FSC range of the bacteria. Data analysis was performed using FlowJo software. Due to the intensive lysis altering the detected signal of opsonized cells (Fig 10), no statistical significance was calculated.

### Detection of C5b-9 (MAC) deposition on *E*. *coli* by flow cytometry

Flow cytometry was used to measure C5b-9-deposition on the surface of *E*. *coli* cells. All steps were the same as described above, except that mouse anti-C5b-9 antibody (A239, Quidel) was used as the primary antibody and Alexa Fluor 647 conjugated rabbit anti-mouse antibody (A21239, Invitrogen) as the secondary antibody. Pore formation was also evaluated via adding propidium iodide (PI) as a DNA stain. The percentage of C5b-9/PI positive cells was determined. The sample preparation steps yielded some PI+ (single positive) cells, but as the percentage of this PI positive population was constant in all samples, it was considered as artifact, and was thereby excluded from data analysis. Single C5b-9 positive cells were present only in very low amounts, suggesting a transient state that would also turn PI positive upon longer incubation.

### Bactericidal tests in NHS and HIS

Bactericidal tests were conducted based on the method of Muschel et al. [67], with modifications. Cells were grown to mid-log phase (OD_600 nm_ ~ 0.5) in P/BE broth, centrifuged, washed in NHS-buffer, and their density was determined *via* measuring the optical density at 600 nm, in Beckmann Coulter DU-7400 spectrophotometer. Cells were diluted in P/BE broth, then 2 x 10^7^ cells were transferred to each test tube. NHS-buffer (control tubes), NHS or HIS (both diluted in NHS-buffer) was added to reach the appropriate final serum concentration. In the experiments testing the effects of exogenously added inhibitors, the serum was pre-incubated with the appropriate inhibitor for 30 minutes at room temperature. The cells were incubated with serum at 37°C for 1h, then cells from the sample tubes were inoculated into 20-fold excess of fresh, serum-free P/BE broth. This dilution ceased complement activation and lowered the concentration of cell debris that could have interfered with the subsequent optical density measurements. After 5h of incubation, optical density (λ = 600 nm) of the samples was measured and compared to those of the control samples.

### Complementation of the ecotin *KO* cells with *E*. *coli* ecotin provided on an IPTG-inducible expression vector

Protecting role of ecotin was tested in complementation experiments. As the two *E*. *coli* strains are not compatible with T7 promoter vectors, we sub-cloned the gene of wild type ecotin from the original pT7-7 [17] vector into the pTFPI-D2-pro-lib [14] phagemid vector using NdeI and XbaI restriction enzyme cleavage sites. The resulting vector, which allows for IPTG-inducible periplasmic expression of ecotin, was named pTac.Eco. The correct sequence of the vector was confirmed by DNS sequencing. Ecotin *KO E*. *coli* ATCC 23505 and ATCC 12014 cells were transformed with the pTac.Eco vector by electroporation. We tested that in P/BE broth, the untransformed cells and the transformed, non-induced cells grew with the same rate, and this rate did not decrease up to a level of 50 μM IPTG added to the transformed cells. Therefore, an IPTG concentration of 50 μM was used for ecotin expression induction in the above described bactericidal tests. Ecotin level in the periplasmic fraction was determined as follows. Wild type *E*. *coli* ATCC 23505 and ATCC 12014 cells, their ecotin *KO* versions, their ecotin *KO* pTac.Eco-transformed but non-induced versions and their ecotin *KO* pTac.Eco-transformed and 50 μM IPTG-induced versions were grown in P/BE broth up to OD_600_ = 1.0. The periplasmic fraction was isolated similarly to the procedure described at the “Purification of *E*. *coli*, *P*. *aeruginosa* and *Y*. *pestis* ecotin orthologs” section in the Materials and methods, but in this case the pelleted cells were resuspended in 10 mL / 1 L culture ice cold dH_2_O and were frozen. The isolated periplasmic fractions were then diluted to the same protein content based on OD_280_ value with 20 mM Tris-HCl, 5 mM CaCl_2_, pH 7.9 buffer. Then, serial dilutions of the buffered periplasmic fractions were mixed with 20 nM final concentration of bovine chymotrypsin (Worthington, #LS001432) and incubated for 1 hour. Free residual enzyme concentration was determined by 250 μM Suc-Ala-Ala-Pro-Phe-PNA substrate (Bachem AG, #1067440).

### Statistical analysis of results

Where statistical significance is indicated, two-tailed unpaired Student’s t-test was performed. Difference between two tested datasets was considered significant if the calculated P value was lower than 0.05.

### Analyzing the phylogenic distribution of ecotin harboring organisms

Species were selected from the *PFAM* database [68] with the keyword „ecotin”. Only species with proper identification were analyzed, isolates such as “*Pseudomonas sp*.” were excluded. Multiplicates representing multiple entries/ecotin domains from the same microorganism were removed. Ecotin-expressing species were considered pathogenic if they are listed in at least one of the following databases: *Global RPH* (globalrph.com/bacteria), *Bode Science Center* (www.bode-science-center.com/center/relevant-pathogens-from-a-z.html), or *American Biological Safety Association* (my.absa.org/Riskgroups). Number of species within individual phylogenic groups was looked up in the data of *Catalogue of Life* (www.catalogueoflife.org/col).

## Supporting information

S1 Table Phylogenic distribution of ecotin-harboring species. (DOCX)
