# Supplementary material for: Ecotin, a microbial inhibitor of serine proteases, blocks multiple complement dependent and independent microbicidal activities of human serum
Source: PLoS Pathog. 2019 Dec 20;15(12):e1008232. doi: 10.1371/journal.ppat.1008232 (PMC6944378; doi:10.1371/journal.ppat.1008232)
Supplement: S1 Table — (DOCX) [file ppat.1008232.s001.docx]

**S Table. Phylogenic distribution of ecotin-harboring species.** Species with at least one ecotin entry were selected from the PFAM database. In each class, relative frequency (RF, in percentage) of pathogens within the ecotin harboring species was compared to that within all species in the same class. In the case of most microbe classes, greater percent of ecotin harboring species are pathogenic than all species suggesting that ecotin provides an adaptive advantage for pathogenicity.

*Pathogenicity was assigned to species if the species is listed in at least one of the following databases: **1.** Global RPH, pathogen database; **2.** Bode Science Center, list of relevant pathogens; **3.** American Biological Safety Association, list of human risk groups.

** SwissProt entries are highlighted in bold.

| **Class** | **RF of pathogens of ecotin harboring species**; RF of pathogens within all species* | **Species with ecotin** | **PFAM entry of ecotin** | **UniProt entry of ecotin**** | **Database*** |
| --- | --- | --- | --- | --- | --- |
| **Kinetoplastida** | **16.7%**; nd | Trypanosoma theileri | A0A1X0NTQ2_9TRYP | A0A1X0NTQ2 |  |
|  |  | Trypanosoma brucei | ECOT2_TRYB2 | **Q57ZQ7** | 2 |
|  |  | Angomonas deanei | S9VEJ0_9TRYP | S9VEJ0 |  |
|  |  | Strigomonas culicis | S9ULP4_9TRYP | S9ULP4 |  |
|  |  | Trypanosoma rangeli | A0A061J5P3_TRYRA | A0A061J5P3 |  |
|  |  | Trypanosoma congolense | F9W391_TRYCI | F9W391 |  |
|  |  | Trypanosoma cruzi | ECOT2_TRYCC | **Q4D4Y5** | 2 |
|  |  | Leptomonas pyrrhocoris | A0A0N0DQP5_9TRYP | A0A0N0DQP5 |  |
|  |  | Leishmania braziliensis | ECOT1_LEIBR | **A4H823** |  |
|  |  | Leishmania infantum | ECOT2_LEIIN | **A4HWE9** |  |
|  |  | Leishmania major | ECOT1_LEIMA | **Q4QFD4** |  |
|  |  | Leptomonas seymouri | A0A0N1HZ51_LEPSE | A0A0N1HZ51 |  |
| **Isochrysidales** | **0%**; nd | Emiliania huxleyi | R1EV50_EMIHU | R1EV50 |  |
| **Mycetozoa** | **0%**; 0% | Polysphondylium pallidum | D3AWF5_POLPP | D3AWF5 |  |
| **Chlorophyta** | **0%**; 0% | Chlamydomonas eustigma | A0A250XGN3_9CHLO | A0A250XGN3 |  |
| **Synechococcales** | **0%**; 0% | Prochlorococcus marinus | ECOT_PROMM | **P59839** |  |
|  |  | Cyanobium gracile | K9PAL2_CYAGP | K9PAL2 |  |
| **Solibacteres** | **0%**; nd | Solibacter usitatus | Q01NS3_SOLUE | Q01NS3 |  |
| **Chitinophagia** | **0%**; 0% | Niabella drilacis | A0A1G6Y928_9BACT | A0A1G6Y928 |  |
|  |  | Chitinophaga eiseniae | A0A1T4M6J3_9BACT | A0A1T4M6J3 |  |
|  |  | Niabella ginsenosidivorans | A0A1A9I2U4_9BACT | A0A1A9I2U4 |  |
| **Sphingobacteriia** | **11.1%**; 2.2% | Sphingobacterium wenxiniae | A0A1I6RXJ7_9SPHI | A0A1I6RXJ7 |  |
|  |  | Sphingobacterium spiritivorum | D7VMD6_9SPHI | D7VMD6 | 3 |
|  |  | Sphingobacterium faecium | A0A1R4KVQ4_9SPHI | A0A1R4KVQ4 |  |
|  |  | Sphingobacterium cellulitidis | A0A235DXN2_9SPHI | A0A235DXN2 |  |
|  |  | Sphingobacterium psychroaquaticum | A0A1X7KIQ0_9SPHI | A0A1X7KIQ0 |  |
|  |  | Sphingobacterium mizutaii | A0A239WQ70_9SPHI | A0A239WQ70 |  |
|  |  | Sphingobacterium paucimobilis | U2HSV5_9SPHI | U2HSV5 |  |
|  |  | Pseudopedobacter saltans | F0SA18_PSESL | F0SA18 |  |
|  |  | Sphingobacterium nematocida | A0A1T5G8Y0_9SPHI | A0A1T5G8Y0 |  |
| **Class** | **RF of pathogens of ecotin harboring species**; RF of pathogens within all species* | **Species with ecotin** | **PFAM entry of ecotin** | **UniProt entry of ecotin**** | **Database*** |
| **Bacteroidia** | **0%**; 8.8% | Porphyromonas catoniae | Z4WUQ6_9PORP | Z4WUQ6 |  |
|  |  | Bacteroides reticulotermitis | W4UYW6_9BACE | W4UYW6 |  |
|  |  | Dysgonomonas gadei | F5IYK4_9BACT | F5IYK4 |  |
|  |  | Dysgonomonas mossii | F8WY14_9BACT | F8WY14 |  |
| **Flavobacteriia** | **8.3%**; 3.7% | Chryseobacterium indologenes | W4T5X9_9FLAO | W4T5X9 | 3 |
|  |  | Chryseobacterium gallinarum | A0A0G3MDB7_9FLAO | A0A0G3MDB7 |  |
|  |  | Myroides marinus | A0A163XC87_9FLAO | A0A163XC87 |  |
|  |  | Chryseobacterium soli | A0A086A155_9FLAO | A0A086A155 |  |
|  |  | Riemerella anatipestifer | J9QSM3_RIEAN | J9QSM3 |  |
|  |  | Elizabethkingia anophelis | A0A077EG67_9FLAO | A0A077EG67 |  |
|  |  | Myroides profundi | A0A0B5RYX7_9FLAO | A0A0B5RYX7 |  |
|  |  | Chryseobacterium taiwanense | A0A0B4D0Q7_9FLAO | A0A0B4D0Q7 |  |
|  |  | Chryseobacterium glaciei | A0A172XYQ3_9FLAO | A0A172XYQ3 |  |
|  |  | Myroides odoratus | H1Z4U0_MYROD | H1Z4U0 | 3 |
|  |  | Lutibacter agarilyticus | A0A238VJ00_9FLAO | A0A238VJ00 |  |
|  |  | Chryseobacterium luteum | A0A085ZCE0_9FLAO | A0A085ZCE0 |  |
|  |  | Chryseobacterium carnipullorum | A0A1M7L3F8_9FLAO | A0A1M7L3F8 |  |
|  |  | Soonwooa buanensis | A0A1T5G625_9FLAO | A0A1T5G625 |  |
|  |  | Chryseobacterium cucumeris | A0A150ZUV2_9FLAO | A0A150ZUV2 |  |
|  |  | Chryseobacterium gambrini | A0A1N7Q3X5_9FLAO | A0A1N7Q3X5 |  |
|  |  | Chryseobacterium arachidis | A0A1M5HD30_9FLAO | A0A1M5HD30 |  |
|  |  | Chryseobacterium artocarpi | A0A1B9A0C9_9FLAO | A0A1B9A0C9 |  |
|  |  | Chishuiella changwenlii | A0A1M6SXA6_9FLAO | A0A1M6SXA6 |  |
|  |  | Algoriella xinjiangensis | A0A1I4XMU6_9FLAO | A0A1I4XMU6 |  |
|  |  | Chryseobacterium piperi | A0A086B4I4_9FLAO | A0A086B414 |  |
|  |  | Chryseobacterium contaminans | A0A1M7DA31_9FLAO | A0A1M7DA31 |  |
|  |  | Chryseobacterium formosense | A0A085Z8I1_9FLAO | A0A085Z8I1 |  |
|  |  | Apibacter mensalis | A0A0X3AN48_9FLAO | A0A0X3AN48 |  |
| **Epsilon proteobacteria** | **55.6%**; 19% | Campylobacter showae | C6RIL7_9PROT | C6RIL7 |  |
|  |  | Campylobacter ureolyticus | S3XIN1_9PROT | S3XIN1 |  |
|  |  | Campylobacter curvus | A7GWV6_CAMC5 | A7GWV6 | 3 |
|  |  | Campylobacter rectus | B9D376_CAMRE | B9D376 | 3 |
|  |  | Campylobacter hominis | A7I1Y3_CAMHC | A7I1Y3 |  |
|  |  | Campylobacter fetus | A0A128EF46_CAMFE | A0A128EF46 | 1, 3 |
|  |  | Campylobacter concisus | A0A0M4TNR1_9PROT | A0A0M4TNR1 | 3 |
|  |  | Campylobacter gracilis | C8PJ18_9PROT | C8PJ18 | 3 |
|  |  | Sulfurovum riftiae | A0A151CGA7_9PROT | A0A151CGA7 |  |

| **Class** | **RF of pathogens of ecotin harboring species**; RF of pathogens within all species* | **Species with ecotin** | **PFAM entry of ecotin** | **UniProt entry of ecotin**** | **Database*** |
| --- | --- | --- | --- | --- | --- |
| **Alpha proteobacteria** | **25%**; 4.2% | Rhizobium favelukesii | W6RT02_9RHIZ | W6RT02 |  |
|  |  | Rhizobium altiplani | A0A109JJM4_9RHIZ | A0A109JJM4 |  |
|  |  | Commensalibacter intestini | G6EYD2_9PROT | G6EYD2 |  |
|  |  | Rickettsia bellii | Q1RHL1_RICBR | Q1RHL1 | 3 |
|  |  | Rickettsia hoogstraalii | A0A0F3PN67_9RICK | A0A0F3PN67 |  |
|  |  | Brevundimonas diminuta | F4R2E7_BREDA | L1QAV4 | 2 |
|  |  | Paracoccus aminophilus | S5XT79_PARAH | S5XT79 |  |
|  |  | Pontibaca methylaminivorans | A0A1R3WF33_9RHOB | A0A1R3WF33 |  |
| **Gamma proteobacteria** | **24.6%**; 10.2% | Pasteurella skyensis | A0A1H7VMW0_9PAST | A0A1H7VMW0 |  |
|  |  | Serratia fonticola | A0A0F7HCX9_SERFO | A0A0F7HCX9 |  |
|  |  | Yersinia nurmii | A0A0T9KMT3_9GAMM | A0A0T9KMT3 |  |
|  |  | Yersinia pestis | ECOT_YERPE | **Q8ZGS0** | 2, 3 |
|  |  | Yersinia mollaretii | C4SD69_YERMO | C4SD69 |  |
|  |  | Serratia plymuthica | A0A1B1KNT9_SERPL | A0A1B1KNT9 |  |
|  |  | Yersinia pekkanenii | A0A0T9QQM2_9GAMM | A0A0T9QQM2 |  |
|  |  | Chania multitudinisentens | W0LDX2_9GAMM | W0LDX2 |  |
|  |  | Serratia oryzae | A0A1S8CJR3_9GAMM | A0A1S8CJR3 |  |
|  |  | Ewingella americana | A0A085G824_9GAMM | A0A085G824 | 3 |
|  |  | Yersinia ruckeri | A0A085U4T4_YERRU | A0A085U4T4 |  |
|  |  | Serratia odorifera | D4E8L4_SEROD | D4E8L4 |  |
|  |  | Serratia ficaria | A0A240BQD2_SERFI | A0A240BQD2 |  |
|  |  | Proteus mirabilis | B4F0H3_PROMH | B4F0H3 | 1, 2, 3 |
|  |  | Xenorhabdus nematophila | D3V9P8_XENNA | D3V9P8 |  |
|  |  | Xenorhabdus khoisanae | A0A0J5FN40_9GAMM | A0A0J5FN40 |  |
|  |  | Xenorhabdus vietnamensis | A0A1Y2S9M9_9GAMM | A0A1Y2S9M9 |  |
|  |  | Cosenzaea myxofaciens | A0A198GE16_9GAMM | A0A198GE16 |  |
|  |  | Xenorhabdus bovienii | D3UXW8_XENBS | D3UXW8 |  |
|  |  | Moellerella wisconsensis | A0A0N0ZAH6_9GAMM | A0A0N0ZAH6 |  |
|  |  | Xenorhabdus hominickii | A0A2G0Q009_9GAMM | A0A2G0Q009 |  |
|  |  | Xenorhabdus stockiae | A0A2D0KLF7_9GAMM | A0A2D0KLF7 |  |
|  |  | Proteus penneri | C0AXJ1_9GAMM | C0AXJ1 | 3 |
|  |  | Xenorhabdus szentirmaii | W1J274_9GAMM | W1J274 |  |
|  |  | Providencia alcalifaciens | W3YGC1_9GAMM | W3YGC1 | 3 |
|  |  | Providencia rettgeri | K8W3B5_PRORD | K8W3B5 | 1, 2, 3 |
|  |  | Xenorhabdus innexi | A0A1N6MVE2_9GAMM | A0A1N6MVE2 |  |
|  |  | Morganella morganii | M1RNJ6_MORMO | M1RNJ6 | 3 |
|  |  | Xenorhabdus doucetiae | A0A068QUA2_9GAMM | A0A068QUA2 |  |
|  |  | Xenorhabdus cabanillasii | W1IQW4_9GAMM | W1IQW4 |  |
|  |  | Xenorhabdus poinarii | A0A068R0P0_9GAMM | A0A068R0P0 |  |
|  |  | Providencia heimbachae | A0A1B7JJ98_9GAMM | A0A1B7JJ98 |  |
| **Class** | **RF of pathogens of ecotin harboring species**; RF of pathogens within all species* | **Species with ecotin** | **PFAM entry of ecotin** | **UniProt entry of ecotin**** | **Database*** |
| **Gamma proteobacteria** | **24.6%**; 10.2% | Xenorhabdus kozodoii | A0A2D0L423_9GAMM | A0A2D0L423 |  |
|  |  | Providencia burhodogranariea | K8WVS4_9GAMM | K8WVS4 |  |
|  |  | Providencia sneebia | K8WEI3_9GAMM | K8WEI3 |  |
|  |  | Xenorhabdus thuongxuanensis | A0A1Q5TTH0_9GAMM | A0A1Q5TTH0 |  |
|  |  | Xenorhabdus beddingii | A0A1Y2SF56_9GAMM | A0A1Y2SF56 |  |
|  |  | Xenorhabdus mauleonii | A0A1I3X6X5_9GAMM | A0A1I3X6X5 |  |
|  |  | Leclercia adecarboxylata | A0A1V2YYK2_9ENTR | A0A1V2YYK2 | 2, 3 |
|  |  | Klebsiella oxytoca | A0A0H3HCN3_KLEOK | A0A0H3HCN3 | 2, 3 |
|  |  | Klebsiella pneumoniae | A6TBS4_KLEP7 | A6TBS4 | 1, 2, 3 |
|  |  | Cronobacter sakazakii | A7MNY6_CROS8 | A7MNY6 |  |
|  |  | Citrobacter rodentium | D2TSC1_CITRI | D2TSC1 |  |
|  |  | Enterobacter lignolyticus | E3GBA1_ENTLS | E3GBA1 |  |
|  |  | Shigella flexneri | ECOT_SHIFL | **Q0T2R6** | 1, 3 |
|  |  | Escherichia coli | ECOT_ECO57 | **B5YX01** | 1, 2, 3 |
|  |  | Klebsiella aerogenes | A0A0H3FYX7_KLEAK | A0A0H3FYX7 | 3 |
|  |  | Gibbsiella quercinecans | A0A250AXL6_9ENTR | A0A250AXL6 |  |
|  |  | Trabulsiella guamensis | A0A085AFK8_9ENTR | A0A085AFK8 |  |
|  |  | Kluyvera georgiana | A0A248KHB3_9ENTR | A0A248KHB3 | 3 |
|  |  | Enterobacter cloacae | G8LKM5_ENTCL | G8LKM5 | 1, 2, 3 |
|  |  | Citrobacter koseri | A8AE08_CITK8 | A8AE08 | 1, 3 |
|  |  | Salmonella typhimurium | ECOT_SALTY | **Q8ZNH4** | 2, 3 |
|  |  | Pluralibacter gergoviae | A0A0J5M0X6_PLUGE | A0A0J5M0X6 |  |
|  |  | Salmonella arizonae | ECOT_SALAR | **A9MJZ3** | 1, 3 |
|  |  | Yokenella regensburgei | G9ZA87_9ENTR | G9ZA87 |  |
|  |  | Raoultella ornithinolytica | A0A0B5IU88_RAOOR | A0A0B5IU88 |  |
|  |  | Citrobacter werkmanii | A0A090UKX7_9ENTR | A0A090UKX7 | 3 |
|  |  | Shimwellia blattae | I2B8G8_SHIBC | I2B8G8 |  |
|  |  | Kluyvera ascorbata | A0A085IF80_9ENTR | A0A085IF80 | 3 |
|  |  | Plesiomonas shigelloides | R8AQ49_PLESH | R8AQ49 | 3 |
|  |  | Leminorella grimontii | A0A085HJ04_9GAMM | A0A085HJ04 |  |
|  |  | Budvicia aquatica | A0A2C6DMQ2_9GAMM | A0A2C6DMQ2 |  |
|  |  | Pragia fontium | A0A0G3CNW7_9GAMM | A0A0G3CNW7 |  |
|  |  | Pantoea gaviniae | A0A1X1DT47_9GAMM | A0A1X1DT47 |  |
|  |  | Tatumella ptyseos | A0A085JCT4_9GAMM | A0A085JCT4 | 3 |
|  |  | Pantoea alhagi | A0A1W6B7N9_9GAMM | A0A1W6B7N9 |  |
|  |  | Tatumella morbirosei | A0A095TA35_9GAMM | A0A095TA35 |  |
|  |  | Edwardsiella ictaluri | C5BEG4_EDWI9 | C5BEG4 | 3 |
|  |  | Hafnia alvei | A0A097R4L6_HAFAL | A0A097R4L6 | 3 |
|  |  | Aeromonas molluscorum | R1F583_9GAMM | R1F583 |  |
|  |  | Aeromonas hydrophila | A0KIU3_AERHH | A0KIU3 | 1, 3 |
| **Class** | **RF of pathogens of ecotin harboring species**; RF of pathogens within all species* | **Species with ecotin** | **PFAM entry of ecotin** | **UniProt entry of ecotin**** | **Database*** |
| **Gamma proteobacteria** | **24.6%**; 10.2% | Aeromonas schubertii | A0A0S2SND4_9GAMM | A0A0S2SND4 | 1, 3 |
|  |  | Aeromonas caviae | A0A125Y0M8_AERCA | A0A125Y0M8 | 3 |
|  |  | Gilliamella bombicola | A0A1C4CN58_9GAMM | A0A1C4CN58 |  |
|  |  | Frischella perrara | A0A0A7S1K1_9GAMM | A0A0A7S1K1 |  |
|  |  | Gilliamella apicola | X2GWX5_9GAMM | X2GWX5 |  |
|  |  | Thalassotalea agarivorans | A0A1H9Y8K6_THASX | A0A1H9Y8K6 |  |
|  |  | Shewanella amazonensis | A1S6F3_SHEAM | A1S6F3 |  |
|  |  | Shewanella morhuae | A0A1N7AV27_9GAMM | A0A1N7AV27 |  |
|  |  | Shewanella pealeana | A8H512_SHEPA | A8H512 |  |
|  |  | Shewanella algae | A0A1S2TWV8_9GAMM | A0A1S2TWV8 | 3 |
|  |  | Shewanella putrefaciens | A4Y711_SHEPC | A4Y711 |  |
|  |  | Shewanella frigidimarina | Q082P3_SHEFN | Q082P3 |  |
|  |  | Shewanella violacea | D4ZLY7_SHEVD | D4ZLY7 |  |
|  |  | Shewanella colwelliana | A0A1E5INY2_SHECO | A0A1E5INY2 |  |
|  |  | Shewanella psychrophila | A0A1S6HMM7_9GAMM | A0A1S6HMM7 |  |
|  |  | Shewanella oneidensis | ECOT_SHEON | **Q8EEQ7** |  |
|  |  | Shewanella sediminis | A8FV10_SHESH | A8FV10 |  |
|  |  | Shewanella denitrificans | Q12N13_SHEDO | Q12N13 |  |
|  |  | Shewanella loihica | A3QEL2_SHELP | A3QEL2 |  |
|  |  | Shewanella woodyi | B1KGU3_SHEWM | B1KGU3 |  |
|  |  | Shewanella benthica | A9DIB6_9GAMM | A9DIB6 |  |
|  |  | Photobacterium jeanii | A0A178K8P6_9GAMM | A0A178KP6 |  |
|  |  | Photobacterium swingsii | A0A0J8V5X8_9GAMM | A0A0J8V5X8 |  |
|  |  | Vibrio campbellii | A7N5L4_VIBCB | A7N5L4 |  |
|  |  | Vibrio proteolyticus | U3BFH5_VIBPR | U3BFH5 |  |
|  |  | Photobacterium gaetbulicola | A0A0C5WZP6_9GAMM | A0A0C5WZP6 |  |
|  |  | Vibrio nigripulchritudo | U4KIS6_9VIBR | U4KIS6 |  |
|  |  | Microbulbifer thermotolerans | A0A143HHP2_9GAMM | A0A143HHP2 |  |
|  |  | Microbulbifer donghaiensis | A0A1M5AYU6_9GAMM | A0A1M5AYU6 |  |
|  |  | Thiothrix eikelboomii | A0A1T4WI96_9GAMM | A0A1T4WI96 |  |
|  |  | Alcanivorax dieselolei | K0CGN3_ALCDB | K0CGN3 |  |
|  |  | Pseudomonas lundensis | A0A1D9IS31_9PSED | A0A1D9IS31 |  |
|  |  | Pseudomonas aeruginosa | ECOT_PSEAE | **Q9I088** | 1, 2, 3 |
|  |  | Pseudomonas mendocina | A4XX51_PSEMY | A4XX51 | 3 |
|  |  | Pseudomonas alkylphenolica | A0A077F929_9PSED | A0A077F929 |  |
|  |  | Pseudomonas knackmussii | A0A024HDA8_PSEKB | A0A024HDA8 |  |
|  |  | Pseudomonas plecoglossicida | S2K380_9PSED | S2K380 |  |
|  |  | Oblitimonas alkaliphila | A0A0K1XEY9_9GAMM | A0A0K1XEY9 |  |
|  |  | Pseudomonas brenneri | A0A176V842_9PSED | A0A176V842 |  |
|  |  | Pseudomonas fluorescens | A0A010SKN8_PSEFL | **Q4KC31** |  |
| **Class** | **RF of pathogens of ecotin harboring species**; RF of pathogens within all species* | **Species with ecotin** | **PFAM entry of ecotin** | **UniProt entry of ecotin**** | **Database*** |
| **Gamma proteobacteria** | **24.6%**; 10.2% | Pseudomonas putida | A0A0D1LYI9_PSEPU | **Q88IC7** |  |
|  |  | Pseudomonas batumici | A0A0C2EXF4_9PSED | A0A0C2EXF4 |  |
|  |  | Pseudomonas chlororaphis | J2YI44_9PSED | J2YI44 |  |
|  |  | Pseudomonas citronellolis | A0A1I1JL56_9PSED | A0A1I1JL56 |  |
|  |  | Pseudomonas agarici | A0A0X1SYE8_PSEAA | A0A0X1SYE8 |  |
|  |  | Pseudomonas fuscovaginae | A0A0N0VKE4_9PSED | A0A0N0VKE4 |  |
|  |  | Pseudomonas endophytica | A0A0Q0XTG0_9PSED | A0A0Q0XTG0 |  |
|  |  | Pseudomonas psychrophila | A0A0J6KPX8_9PSED | A0A0J6KPX8 |  |
|  |  | Acinetobacter guillouiae | N8TAL9_ACIGI | N8TAL9 |  |
|  |  | Acinetobacter rudis | S3N4H0_9GAMM | S3NCT7 |  |
| **Beta proteobacteria** | **6.3%**; 6% | Kerstersia gyiorum | A0A171KTL1_9BURK | A0A171KTL1 |  |
|  |  | Paenalcaligenes hominis | A0A1U9K1D3_9BURK | A0A1U9K1D3 |  |
|  |  | Advenella mimigardefordensis | W0P5Q7_9BURK | W0P5Q7 |  |
|  |  | Burkholderia cenocepacia | B4E732_BURCJ | B4E732 |  |
|  |  | Burkholderia vietnamiensis | A4JGS2_BURVG | A4JGS2 |  |
|  |  | Burkholderia multivorans | A0A0H3KQE2_BURM1 | A0A0H3KQE2 |  |
|  |  | Burkholderia ubonensis | A0A125JX75_9BURK | A0A125JX75 |  |
|  |  | Burkholderia pseudomallei | Q63W37_BURPS | Q63W37 | 1, 2, 3 |
|  |  | Burkholderia ambifaria | B1TDR8_9BURK | B1TDR8 |  |
|  |  | Burkholderia gladioli | F2LFQ7_BURGS | F2LFQ7 |  |
|  |  | Burkholderia glumae | C5ABE8_BURGB | C5ABE8 |  |
|  |  | Herminiimonas arsenicoxydans | A4G371_HERAR | A4G371 |  |
|  |  | Thauera linaloolentis | N6YPB4_9RHOO | N6YPB4 |  |
|  |  | Accumulibacter phosphatis | C7RM46_ACCPU | C7RM46 |  |
|  |  | Methylobacillus rhizosphaerae | A0A239B5D7_9PROT | A0A239B5D7 |  |
|  |  | Methylobacillus flagellatus | ECOTL_METFK | **Q1H1S3** |  |
| **Delta proteobacteria** | **0%**; 0% | Sorangium cellulosum | S4XTU7_SORCE | S4XTU7 |  |
|  |  | Desulfopila aestuarii | A0A1M7YJD5_9DELT | A0A1M7YJD5 |  |
|  |  | Desulfoluna spongiiphila | A0A1G5IS06_9DELT | A0A1G5IS06 |  |
| **Fusobacteriales** | **100%**; 42.4% | Fusobacterium necrophorum | H1D981_9FUSO | H1D981 | 1, 3 |
|  |  | Fusobacterium gonidiaformans | E5BF37_9FUSO | E5BF37 | 3 |
| **Planctomicetida** | **0%**; 0% | Planctopirus limnophila | D5SQK2_PLAL2 | D5SQK2 |  |
| **Verrucimicrobiae** | **0%**; 0% | Rubritalea squalenifaciens | A0A1M6EF40_9BACT | A0A1M6EF40 |  |
